# Synthesis and In Vitro Biocompatibility Studies of Novel Alkoxy 4,4-Difluoro-4-bora-3a,4a-diaza-s-indacenes

**DOI:** 10.3390/ma16227085

**Published:** 2023-11-08

**Authors:** Margarida G. Santos, Juliana Araújo, Chrislaura Carmo, Leonardo Santos, Maria Filomena Botelho, Mafalda Laranjo, Abílio J. F. N. Sobral

**Affiliations:** 1 Department of Physics, University of Coimbra, 3004-516 Coimbra, Portugal; goncalvesmagy@gmail.com; 2 Department of Chemistry, University of Coimbra, 3004-535 Coimbra, Portugal; juliana.g.araujo2304@gmail.com (J.A.); cribego@gmail.com (C.C.);; 3 Coimbra Institute for Clinical and Biomedical Research (iCBR), Area of Environment, Genetics and Oncobiology (CIMAGO), and Institute of Biophysics, Faculty of Medicine, University of Coimbra, 3000-354 Coimbra, Portugal; mfbotelho@fmed.uc.pt (M.F.B.); mafaldalaranjo@gmail.com (M.L.); 4 Center for Innovative Biomedicine and Biotechnology (CIBB), University of Coimbra, 3000-548 Coimbra, Portugal; 5 Clinical Academic Centre of Coimbra (CACC), 3000-354 Coimbra, Portugal

**Keywords:** BODIPY, biocompatibility, characterisation, applications

## Abstract

BODIPYs are bicyclic aromatic compounds with unique spectroscopic, photophysical, and chemical properties. This study aimed to find BODIPYs with characteristics biocompatible with human cell lines for possible use as imaging agents. Six BODIPY derivatives were synthesised with groups linked to boron, fluorine, phenol, or catechol, resulting in compounds with different physicochemical characteristics. NMR, absorption, and emission spectroscopy and mass spectrometry were subsequently used to characterise them. Afterwards, the biocompatibility of these compounds was evaluated using MTT, SRB, and cellular uptake assays in A549 and H1299 cell lines. Furthermore, a haemolysis assay was performed on human blood cells. To summarise the main results, BODIPYs 1 to 4 showed considerable fluorescence. In contrast, BODIPYs 5 and 6 showed very weak fluorescence, which could be related to the presence of the catechol group and its quenching properties. Regarding biocompatibility, all compounds had metabolic activity and viability above 80% and 70%, respectively. BODIPYs 3 and 6 presented the most consistent data, demonstrating good uptake and, in general, haemolytic activity below 25%. In conclusion, the cytotoxic effects of the compounds were not considerable, and the presence of cyclic alkoxides in BODIPYs 3 and 6 may introduce exciting features that should be highlighted for dual imaging for BODIPY 3 due to its fluorescence or for radioactive labelling in the case of both BODIPYs.

## 1. Introduction

BODIPYs, or 4,4-difluoro-4-bora-3a,4a-diaza-s-indacenes, are small aromatic compounds with interesting properties. These compounds absorb high amounts of ultraviolet (UV) and generate relatively strong fluorescence peaks [1]. Their spectroscopic, photophysical, and chemical characteristics can be defined by the fluorophores’ extensive structural and substitutive versatility [2,3,4,5,6]. Additionally, they possess desirable features such as photochemical stability, narrow absorption and emission bands with intense peaks, a high molar absorption coefficient ε(λ), a high quantum fluorescence yield (Φ), and a fluorescence time (τ) in the nanosecond range [2,3,4,6,7,8]. In addition to their optical properties, BODIPY compounds are known for their low toxicity, thermal stability, and resistance to self-aggregation in solution under physiological conditions [2,6,7]. Their solubility in various organic solvents remains unaffected by the surrounding environment’s polarity and pH [1,2,4,5,6,7,9].

BODIPYs find applications in numerous fields and are used as fluorescent sensors, photoactive materials in organic photovoltaic devices, organic light-emitting devices, fluorescent nanocarriers, photoactivatable compounds, and photocatalysts in organic reactions, among other applications [2,4,5,6,7,8,10]. Furthermore, these versatile compounds are commercially available as boron dipyrromethene dyes that find wide usage as biological labels, bioimaging probes, and laser dyes [6]. BODIPY and BODIPY-derived functional materials have been investigated for their use as antibacterial agents, for labelling biological substances or targeting different biomolecules, and for functionalising drug micro- and nanocarriers in medical diagnostics [6,8]. BODIPYs, in combination with fluorine-18, have gained increasing popularity for PET studies in recent years. Some research in the field, such as brain and myocardial perfusion imaging and PET/fluorescence imaging, has previously been published [5,11,12,13,14,15,16].

**Scheme 1:**
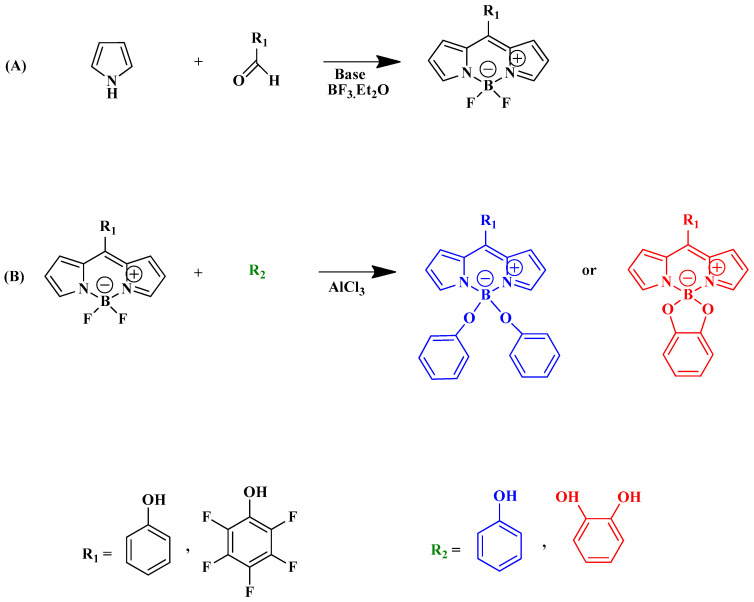
General procedure for synthesising (**A**) BODIPYs 1 and 2 and (**B**) BODIPYs 3 to 6, adapted from [17].

**Scheme 2:**
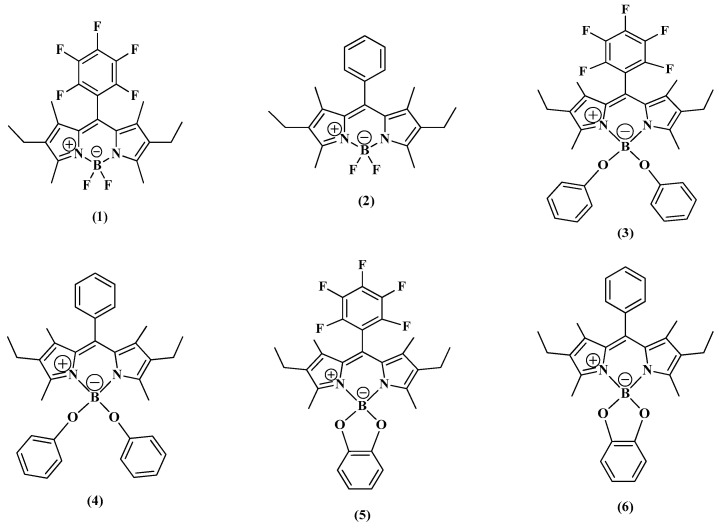
BODIPYs (1)–(6) synthesised.

Six compounds were synthesised, as outlined in Scheme 1. A structural evaluation of the new alkoxy-BODIPYs versus the non-alkoxy-BODIPYs was performed. Additionally, the biocompatibility of BODIPYs exhibiting different planar and non-planar characteristics, such as those represented in Scheme 2, was evaluated.

## 2. Materials and Methods

All reagents, silica gel, and solvents were supplied by Sigma-Aldrich, Acros Organics, or Fluka and used without further purification. Analytical thin-layer chromatography (TLC) was performed on Merck silica gel plates with the indicator F-254, and the results were observed with ultraviolet light (at 254 and 365 nm). Silica gel (230–400 mesh) was used for column chromatography, and Fluka 60G silica gel was used to perform TLC on 20 × 20 cm glass plates.

^1^H, ^13^C, ^11^B, and ^19^F NMR spectra were obtained with a Bruker AVANCE III NMR at 400 MHz, 100 MHz, 128 MHz, and 376 MHz, respectively. As internal standards, protons from the solvent, CDCl_3_, were used. Mass spectra were acquired with an ion trap MS (THERMO SCIENTIFIC, Waltham, MA, USA, model LXQ) employing Electronic Spray Ionisation (ESI), and the samples were dissolved in dichloromethane and diluted in methanol acidified with 0.1% formic acid. The absorption spectra were obtained with a Shimadzu UV-2600 spectrophotometer, with a wavelength window between 200 and 800 nm, using 1 cm path-length quartz cells.

Steady-state fluorescence studies were conducted using a Horiba-Jobin-Yvon Fluorog spectrometer. Spectra were obtained using optically diluted solutions in 1 cm path-length quartz cells.

### 2.1. Synthesis of BODIPYs 1 and 2

In a two-necked flask, nitrogen gas was bubbled into 20 mL of CH_2_Cl_2_ for 5 min. Then, 0.3 mL of 3-ethyl-2,5-dimethyl-pyrrole (2.22 mmol), 50 μL of TFA (0.65 mmol), and 0.97 mmol of the corresponding aldehyde (pentafluorbenzaldehyde and benzaldehyde for BODIPYs 1 and 2, respectively) were added. The mixture was stirred under a nitrogen atmosphere for 10 min. Ten minutes later, 0.27 g of DDQ (1.19 mmol) was added to the reaction mix. Another ten minutes later, 4 mL of triethylamine (28.7 mmol) and 5 mL of BF_3_.Et_2_O (39.8 mmol) were added and stirred for 15 min. The reaction mixture was washed with a saturated aqueous solution of NaHCO_3_ (30 mL), a saturated aqueous solution of NaCl (30 mL), and distilled water (30 mL). The layers were separated, and the organic phase was dried with anhydrous Na_2_SO_4_ and concentrated under reduced pressure.

The BODIPYs were isolated using column chromatography utilising CH_2_Cl_2_–hexane (1:1) and preparative thin-layer chromatography. BODIPYs 1 and 2 were synthesised and used as precursors in the synthesis of BODIPYs 3 to 6.

BDP 1: Yield: 20%; ^1^H NMR (400 MHz, CDCl_3_) δ (ppm): 2.54 (s, 6H), 2.33 (q, J = 7.6 Hz, 4H), 1.51 (s, 6H), 1.02 (t, J = 7.6 Hz, 6H); ^13^C NMR (100 MHz, CDCl_3_) δ (ppm): 156.14, 136.57, 133.93, 130.91, 130.35, 128.83, 121.16, 110.19, 17.08, 14.53, 12.73, 10.84; ^11^B NMR (128 MHz, CDCl_3_) δ (ppm): 0.70 (t, J = 32.64 Hz); ^19^F NMR (376 MHz, CDCl_3_) δ (ppm): −139.25 (dd, J = 15.04 Hz, 7.14 Hz, 2F), −145.61 (dd, J = 32.71 Hz, 2F), −151.06 (t, J = 20.68 Hz, F), −159.82 (dt, J = 21.81, 6.40 Hz, 2F); MS *m*/*z* [M+H]^+^ calculated for C_23_H_22_BF_7_N_2_^+^: 471.23; found: 471.30.

BDP2: Yield: 43%; ^1^H NMR (400 MHz, CDCl_3_) δ (ppm): 7.71–7.70 (m, 2H), 7.54–7.52 (m, 2H), 2.54 (s, 6H), 2.33 (q, J = 7.6 Hz, 4H), 1.51 (s, 6H), 1.02 (t, J = 7.6 Hz, 6H); ^13^C NMR (100 MHz, CDCl_3_) δ (ppm): 156.14, 136.57, 133.92, 130.91, 128.83, 26.61, 17.09, 14.54, 12.74, 10.85; ^11^B NMR (128 MHz, CDCl_3_) δ (ppm): 0.70 (t, J = 32.64 Hz); ^19^F NMR (376 MHz, CDCl_3_) δ (ppm): −145.79 (dd, J = 33.84 Hz, 2F); MS *m*/*z* [M+H]^+^ calculated for C_23_H_28_BF_2_N_2_^+^: 381.28; found: 381.30.

### 2.2. Synthesis of BODIPYs 3 to 6

For the synthesis of BODIPYs 3 to 6, 10 mL of dichloromethane, 0.066 g of AlCl_3_ (0.50 mmol), and 0.050 g of the respective BODIPY 1 (0.11 mmol) or BODIPY 2 (0.13 mmol) were placed in a 100 mL flask. The mixture was refluxed under a N_2_ atmosphere for 15 min at 60 °C in a paraffin bath.

Then, 2.5 equivalents of the respective alcohol reagent—phenol for BODIPYs 3 and 4 and catechol for BODIPYs 5 and 6—was added to the purple solution. The mixture reaction was refluxed and stirred for 20 min. The products were washed with 30 mL of distilled water and 30 mL of saturated aqueous NaCl solution. The organic phase was isolated and dried with anhydrous Na_2_SO_4_. Preparative thin-layer chromatography was performed with hexane–CH_2_Cl_2_ in a proportion of 1:1 to isolate the BODIPYs.

BDP3: Yield: 69%; ^1^H NMR (400 MHz, CDCl_3_) δ (ppm): 7.06 (t, J = 7.2 Hz, 4H), 6.76 (t, J = 7.2 Hz, 2H), 6.65 (d, J = 7.6 Hz, 4H), 2.51 (s, 6H), 2.20 (q, J = 7.6 Hz, 4H), 1.52 (s, 6H), 0.86 (t, J = 7.6 Hz, 6H); ^13^C NMR (100 MHz, CDCl_3_) δ (ppm): 157.01, 156.48, 136.34, 134.50, 130.97, 129.10, 119.50, 118.40, 115.22, 17.08, 14.47, 13.05, 11.02; ^11^B NMR (128 MHz, CDCl_3_) δ (ppm): 0.83 (s); ^19^F NMR (376 MHz, CDCl_3_) δ (ppm): −140.08 (dd, J = 22.56 Hz, 7.52 Hz, 2F), −151.02 (t, J = 20.68 Hz, 1F), −159.79 (dt, J = 21.06 Hz, 7.52 Hz, 2F); MS *m*/*z* [M+H]^+^ calculated for C_35_H_33_BF_5_N_2_O_2_^+^: 619.44; found: 641.20.

BDP4: Yield: 59%; ^1^H NMR (400 MHz, CDCl_3_) δ (ppm): 7.50–7.48 (m, 3H), 7.24–7.22 (m, 2H), 7.08 (t, J = 7.6 Hz, 4H), 6.77 (t, J = 7.2 Hz, 2H), 6.63 (d, J = 7.6 Hz, 4H), 2.52 (s, 6H), 2.19 (q, J = 7.6 Hz, 4H), 1.26 (s, 6H), 0.85 (t, J = 7.6 Hz, 6H); ^13^C NMR (100 MHz, CDCl_3_) δ (ppm): 156.90, 154.48, 138.10, 136.00, 133.29, 131.38, 128.92, 128.66, 128.39, 119.34, 118.80, 17.05, 14.52, 12.84, 11.73; ^11^B NMR (128 MHz, CDCl_3_) δ (ppm): 0.91 (s); MS *m*/*z* [M+H]^+^ calculated for C_35_H_38_BN_2_O_2_^+^: 529.49; found: 551.30.

BDP5: Yield: 64%; ^1^H NMR (400 MHz, CDCl_3_) δ (ppm): 6.76 (s, 4H), 2.27 (q, J = 7.6 Hz, 4H), 2.05 (s, 6H), 1.51 (s, 6H), 0.96 (t, J = 7.6 Hz, 6H); ^13^C NMR (100 MHz, CDCl_3_) δ (ppm): 157.99, 151.96, 137.30, 134.57, 131.05, 119.71, 108.84, 17.09, 14.58, 12.92, 11.03; ^11^B NMR (128 MHz, CDCl_3_) δ (ppm): 7.15 (s); ^19^F NMR (376 MHz, CDCl_3_) δ (ppm): −139.16 (dd, J = 22.56 Hz, 7.52 Hz, 2F), −151.09 (t, J = 22.56 Hz, 1F), −159.83 (dt, J = 22.56 Hz, 7.52 Hz, 2F); MS *m*/*z* [M+H]^+^ calculated for C_29_H_27_BF_5_N_2_O_2_^+^: 541.33; found: 541.30.

BDP6: Yield: 57%; ^1^H NMR (400 MHz, CDCl_3_) δ (ppm): 7.49–7.47 (m, 3H), 7.31–7.29 (m, 2H), 6.78 (s, 4H), 2.23 (q, J = 7.6 Hz, 4H), 2.24 (s, 6H), 1.27 (s, 6H), 0.91 (t, J = 7.6 Hz, 6H); ^13^C NMR (100 MHz, CDCl_3_) δ (ppm): 155.60, 155.41, 151.99, 140.04, 139.18, 136.03, 133.40, 131.49, 129.06, 128.73, 128.33, 128.26, 119.39, 108.74, 17.07, 14.60, 12.69, 11.81; ^11^B NMR (128 MHz, CDCl_3_) δ (ppm): 7.14 (s); MS *m*/*z* [M+H]^+^ calculated for C_29_H_32_BN_2_O_2_^+^: 451.38; found: 451.40.

### 2.3. Cell Culture Conditions

The A549 (ATCC CCL-185) human alveolar basal epithelial lung adenocarcinoma and NCI-H1299 (ATCC CRL-5803) human non-small-cell lung carcinoma cell lines were purchased from the American Type Culture Collection. All cell lines were cultured according to standard procedures at 37 °C in a humidified incubator with 95% air and 5% CO_2_. Cell lines were expanded using Dulbecco’s Modified Eagle medium (DMEM, Gibco^®^, 11966-025), supplemented with 5% heat-inactivated foetal bovine serum (FBS, Sigma F7524, Kawasaki-shi, Japan) for H1299 and 10% FBS for A549 cell line, 1% penicillin–streptomycin (100 U/mL penicillin and 10 mg/mL streptomycin, Lonza Pen Strep, Amphotericin, B, 17-745E), and 0.25 mM sodium pyruvate (Sigma, S8636). For all studies, cells were detached using a solution of 0.25% trypsin-EDTA (Gibco). The Trypan Blue assay was employed to assess cell viability, and cells were only studied when viability exceeded 90%.

The BODIPY solutions to be studied in the biocompatibility studies were prepared using dimethylsulfoxide (DMSO, Sigma, D4540) as a solvent and tested at concentrations ranging from 1 to 100 μM.

#### 2.3.1. MTT Test

Metabolic activity was evaluated using the MTT colourimetric assay (Sigma M2128; Sigma-Aldrich, Inc., Saint Louis, MO, USA) to examine the sensitivity of the cell lines to the BODIPYs. Cell culture plates were washed and then incubated for at least 4 h in the dark at 37 °C with a 3-(4,5-dimethylthiazol-2-yl)-2,5-diphenyltetrazolium bromide (0.5 mg/mL, Sigma M5655) solution in PBS at a pH of 7.4. A 0.04 M hydrochloric acid solution (Merck Millipore100317, Burlington, MA, USA) in isopropanol (Sigma 278475) was added to dissolve the formazan crystals. An EnSpire Multimode Plate Reader (Perkin Elmer, Waltham, MA, USA) was used to measure absorbance at 570 and 620 nm. The percentage ratio between cells treated with BODIPYs and cells treated with the solvent was used to measure metabolic activity.

#### 2.3.2. SRB Test

After the cell cultures had been incubated with the conditioned medium for 24, 48, or 72 h, the sulforhodamine B (SRB) assay was executed. The medium was removed, and the cells were washed with PBS and then fixed for 1 h at 2 °C in a 1% acetic acid solution in methanol. After that, the cells were dyed with a solution of 0.5% SRB in 1% acetic acid, and the plates were incubated for 60 min at room temperature without light. Subsequently, a 10 mM Tri-NaOH solution (pH = 10) was added and homogenised. The EnSpire Multimode Plate Reader (Perkin Elmer, Shelton, CT, USA) was used to read the absorbance at 540 and 690 nm. The ratio between cells treated with BODIPYs and samples treated with the solvent was used to calculate the protein content percentage.

#### 2.3.3. Haemolysis Test

Blood collection tubes were used to collect human blood from healthy donors. First, 1 mL of blood was taken, and the samples were then diluted in 30 volumes of 0.85% NaCl and 10 mM CaCl_2_ solution. After incubation, each tube was filled with diluted blood, which was then adequately mixed by inversion. Afterwards, samples and controls underwent a 1 h incubation period at 37 °C. The tubes containing the samples and the controls underwent centrifugation at 1500× *g* for 3 min after incubation. After collecting the supernatant, 2% of the pellet was resuspended in 5 mL of 0.85% NaCl containing 10 mM CaCl_2_. Next, 96-well plates were filled with 200 μL aliquots. The plates were incubated at room temperature for one and two hours while stirring. Each plate was then centrifuged for 5 min at 4000× *g*. The supernatant was transferred to a second 96-well plate (SPL Life Sciences, 30096, Pocheon-si, Republic of Korea), and the amount of haemoglobin released was measured using the EnSpire Multimode Plate Reader (Perkin Elmer) by measuring the absorbance at 540 nm.

The percentage of released haemoglobin was calculated using the ratio between cells treated with BODIPYs and the difference between controls (cells treated with Triton X-100 and cells treated with DMSO).

#### 2.3.4. Cellular Uptake

Cells (5 × 10^5^) were incubated with BODIPYs 3 and 6 for 1, 2, 4, or 24 h at concentrations of 1 and 10 µM, respectively. Afterwards, cells were PBS-washed and DMSO-disrupted. To accomplish complete disaggregation, cell scrapers were used. The solutions were collected and centrifuged at 13,500× *g*. Fluorescence emission spectroscopy was used to measure the supernatant fluorescence intensity using an EnSpire Multimode Plate Reader (Perkin Elmer). The fluorescence intensity in DMSO solutions for each BODIPY was used to create a calibration curve to calculate the intracellular concentration.

### 2.4. Statistical Analysis

The statistical analysis was performed using Graph Pad Prism version 8.4.3 for Windows, Graph Pad Software, USA. Initially, a Shapiro–Wilk test was performed, and then a parametric test was selected. A one-sample *t*-test was employed, with the maximum normalisation value set at 100% [18]. Multiple comparisons were corrected with the Bonferroni–Dunn test. Values with *p* < 0.05 or lower were considered significant. MTT, SRB, and haemolysis data are represented as means and Confidence Intervals (Cis).

## 3. Results and Discussion

BODIPYs 1 and 2, as presented in Scheme 1A, were synthesised using the standard method, starting with the acid-catalysed condensation of aromatic aldehydes and α-free-pyrroles, followed by a DDQ oxidation step, and concluding with complexation with boron trifluoride in the presence of a base [17]. BODIPYs 3 and 4 were synthesised via the acid-catalysed condensation of phenol and BODIPYs 1 and 2, respectively. To synthesise BODIPYs 5 and 6, catechol was used instead of phenol.

All six BODIPYs were characterised by NMR and mass spectrometry. In addition, absorption, excitation, and emission spectra were obtained. The proton NMR spectra correctly identified the methyl groups and aromatic protons. Carbon spectra revealed the predicted signals for each chemical structure, and the boron and fluorine NMR spectra validated the structure by displaying all of the required and anticipated signals for each molecule. Mass spectra revealed the molecular ion or the molecular ion plus sodium ion.

### 3.1. BODIPY Characterisation

**Figure 1:**
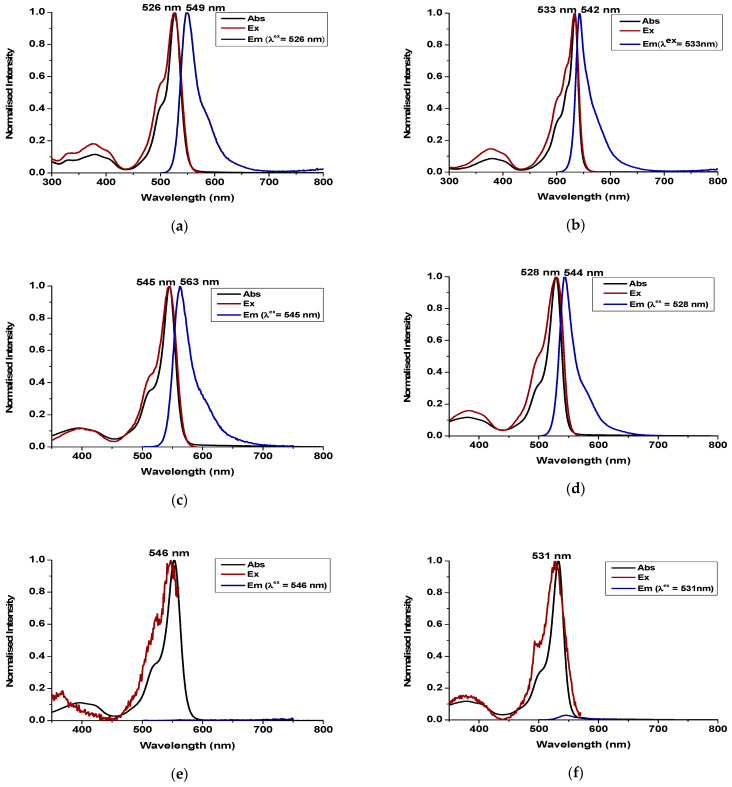
Absorption, excitation, and emission spectra of BODIPYs 1 to 6 in hexane, charts (**a**–**f**).

The absorption, excitation, and emission spectra shown in Figure 1 clearly illustrate the excitation wavelengths and maximum emission peaks for BODIPYs 1 to 4. However, the last two structures studied, BODIPYs 5 and 6, exhibited weak fluorescence. It is likely that this weak fluorescence is quenched by the catechol group, which is the main similarity between these two compounds, as previously observed with other BODIPYs in which the catechol group is covalently linked to the boron atom [17,19,20,21].

On the one hand, the transitions between 300 and 400 nm in the absorbance spectrum may be related to S0->S2 (π-π*) transitions, as reported in the literature [1,22]. The S0->S2 (π-π*) transition could be influenced by the meso-substituted groups, as previously reported in the literature. On the other hand, all compound spectra exhibit a strong S0->S1 (π-π*) transition between 526 and 546 nm with an increased energy shoulder.

The emission tendency displays considerable shifts, which may be related to the sensitivity of BODIPY fluorescence to the rotation of the substituents, especially at the meso-position, and electron transfer [1,22]. The absorption spectra of the molecular thin films show a bathochromic shift of the absorption maximum, likely due to the interactions between adjacent molecules, possibly involving π-orbitals.

### 3.2. Structural Analysis

Bond angles and plane deviations are valuable tools for understanding the molecular properties of the BODIPYs studied. The geometry optimisation of all structures was performed by energy minimisation using MM2 (molecular mechanics) with a minimum RMS (Root Mean Squared) gradient of 0.01 in Chem3D software (CambridgeSoft v12, Yusuf Hamied Department of Chemistry, Cambridge, UK). An assessment of the N-B-N angles and the boron atom planarity with respect to the plane of the pyrrole rings for each structure is summarised in Table 1.

The addition of alkoxy groups to the structure of BODIPYs induced the spatial rearrangement of the compounds, triggering changes in the N-B-N amplitudes, with the alkoxy groups adding extra angle strain to the N-B-N angle [20,23].

The steric effects of the meso-phenyls may have induced differences in planarity between BODIPYs 1 and 2. The deviations observed in BODIPYs 3 to 6 compared to BODIPYs 1 and 2 might be attributed to the structural rearrangement due to the volume of the substituent groups. It is worth noting that the deviations in BODIPYs with catechol groups may indicate a strained region resulting from the catechol addition [19,20,23].

**Figure 2:**
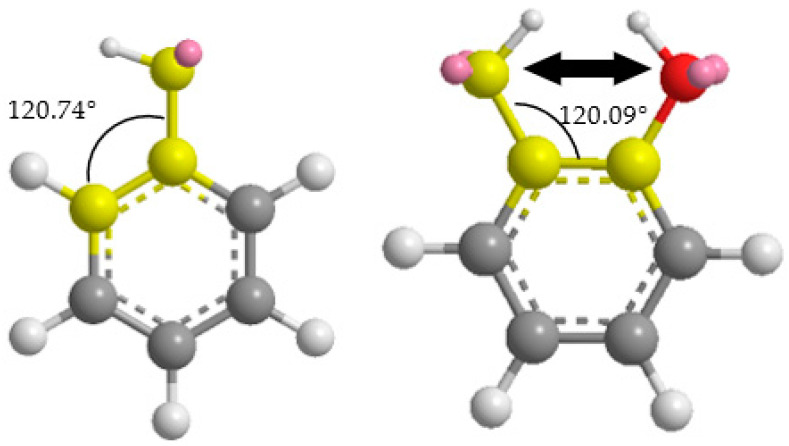
C-C-O angle analysed alone and in BODIPYs.

The C-C-O angles were also considered, either in the phenol and catechol groups alone or in the synthesised compounds. The angle was 120.74° in phenol alone, and there was a slight increase in the angle amplitude after the phenol insertion, which may be related to the volume of the groups added and to the steric effect. The C-C-O angle in catechol alone was 120.09°, as illustrated in Figure 2, indicating additional strain in the oxygen region. The effects of the phenol and catechol group insertions in the boron centre have been extensively studied in other works, and this article provides a straightforward explanation of the structural analysis conducted [19,20,21,22,23].

BODIPYs 5 and 6 exhibited a more pronounced strained region compared to BODIPYs 3 and 4, possibly making them more suitable for labelling, such as in future bioimaging applications [11,12,13,14,15]. Even so, all structures underwent biocompatibility assessments through in vitro studies.

### 3.3. In Vitro Studies

**Figure 3:**
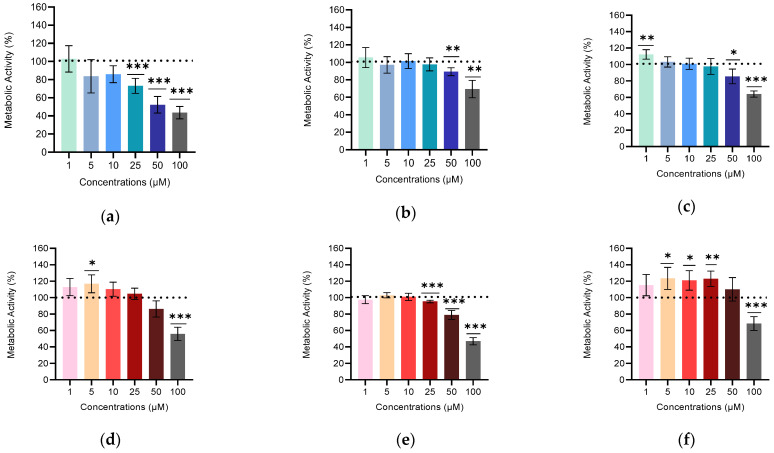
MTT results for BODIPY 1 for the A549 cell line after (**a**) 24 h, (**b**) 48 h, and (**c**) 72 h and for the H1299 cell line after (**d**) 24 h, (**e**) 48 h, and (**f**) 72 h. The results are presented as the mean and CI of a minimum of three independent assays. Statistically significant differences from the control or between conditions are represented by *, where * means *p* < 0.05, ** means *p* < 0.01, and *** means *p* < 0.001.

**Figure 4:**
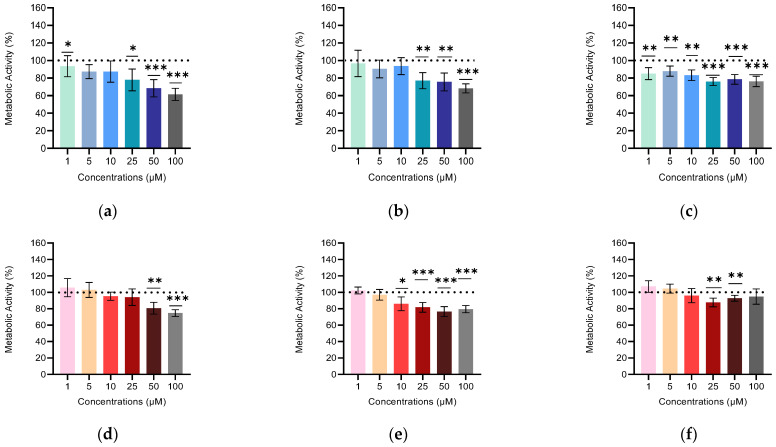
MTT results for BODIPY 2 for the A549 cell line after (**a**) 24 h, (**b**) 48 h, and (**c**) 72 h and for the H1299 cell line after (**d**) 24 h, (**e**) 48 h and (**f**) 72 h. The results are presented as the mean and CI of a minimum of three independent assays. Statistically significant differences from the control or between conditions are represented by *, where * means *p* < 0.05, ** means *p* < 0.01, and *** means *p* < 0.001.

**Figure 5:**
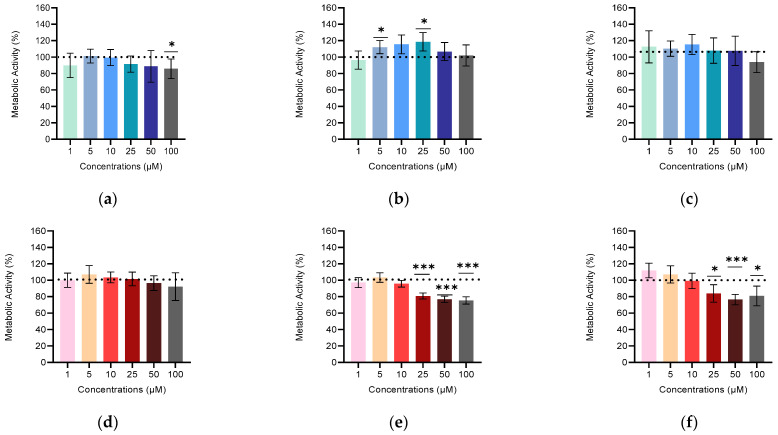
MTT results for BODIPY 3 for the A549 cell line after (**a**) 24 h, (**b**) 48 h, and (**c**) 72 h and for the H1299 cell line after (**d**) 24 h, (**e**) 48 h, and (**f**) 72 h. The results are presented as the mean and CI of a minimum of three independent assays. Statistically significant differences from the control or between conditions are represented by *, where * means *p* < 0.05, and *** means *p* < 0.001.

**Figure 6:**
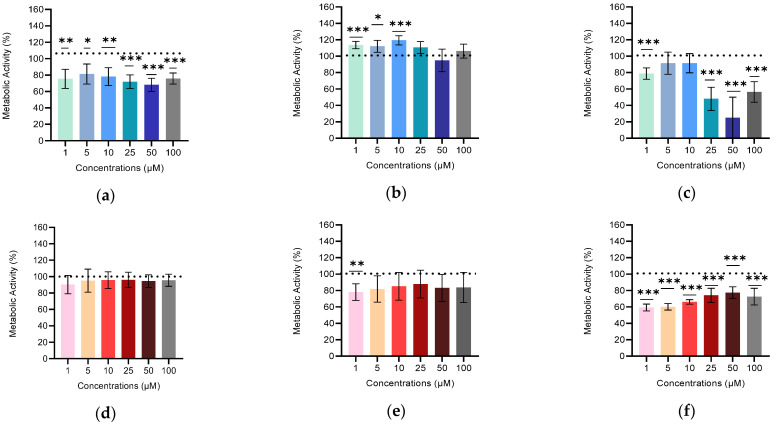
MTT results for BODIPY 4 for the A549 cell line after (**a**) 24 h, (**b**) 48 h, and (**c**) 72 h and for the H1299 cell line after (**d**) 24 h, (**e**) 48 h, and (**f**) 72 h. The results are presented as the mean and CI of a minimum of three independent assays. Statistically significant differences from the control or between conditions are represented by *, where * means *p* < 0.05, ** means *p* < 0.01, and *** means *p* < 0.001.

**Figure 7:**
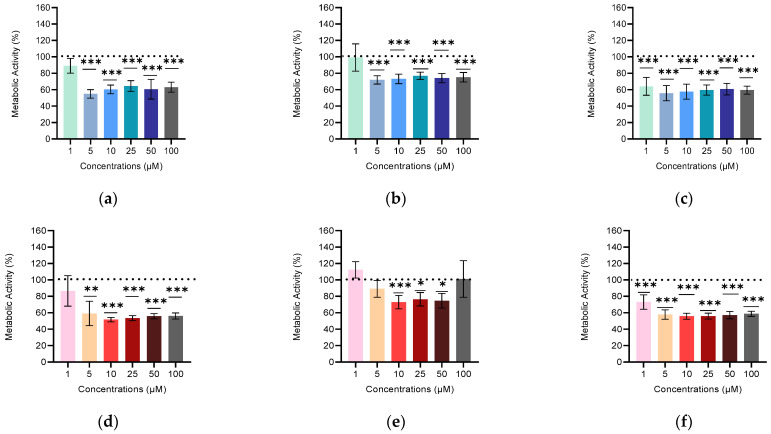
MTT results for BODIPY 5 for the A549 cell line after (**a**) 24 h, (**b**) 48 h, and (**c**) 72 h and for the H1299 cell line after (**d**) 24 h, (**e**) 48 h, and (**f**) 72 h. The results are presented as the mean and CI of a minimum of three independent assays. Statistically significant differences from the control or between conditions are represented by *, where * means *p* < 0.05, ** means *p* < 0.01, and *** means *p* < 0.001.

**Figure 8:**
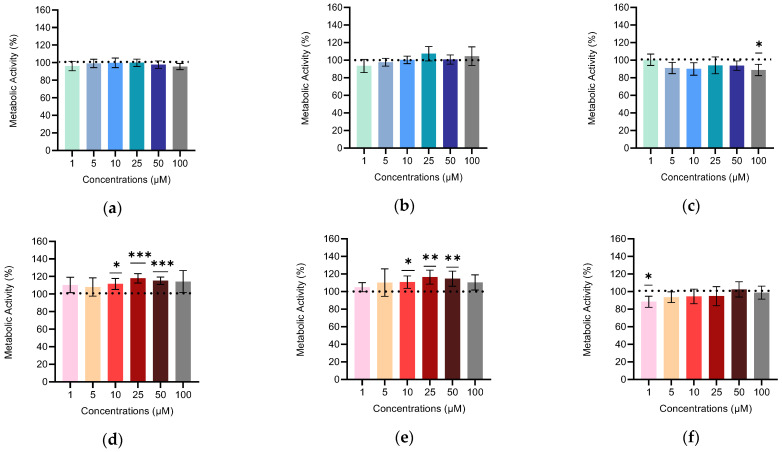
MTT results for BODIPY 6 for the A549 cell line after (**a**) 24 h, (**b**) 48 h, and (**c**) 72 h and for the H1299 cell line after (**d**) 24 h, (**e**) 48 h, and (**f**) 72 h. The results are presented as the mean and CI of a minimum of three independent assays. Statistically significant differences from the control or between conditions are represented by *, where * means *p* < 0.05, ** means *p* < 0.01, and *** means *p* < 0.001.

It is reasonable to infer that higher concentrations of the compounds tend to have a more substantial impact on the decline in the metabolic activity of A549 and H1299 cells, as indicated by the findings and representations in Figure 3, Figure 4, Figure 5, Figure 6, Figure 7 and Figure 8. BODIPY 5 appeared to be related to lower percentages of metabolic activity. Even though BODIPYs 1 and 2 had simpler structures and a strong bond in the boron core, they still exhibited metabolic activity above 80% and viability above 70%. The SRB test was used to confirm which chemicals had less impact on cell viability.

In other studies investigating BODIPYs for lung cancer diagnosis through Nuclear Magnetic Resonance, when their metabolic activity was assessed at concentrations similar to those used in this study, the observed metabolic activity values were above 90% [23]. In comparison, in this study, the data obtained for BODIPYs 3 and 6 at concentrations between 1 µM and 10 µM showed values above 80%, which are promising results for future biomedical applications.

**Figure 9:**
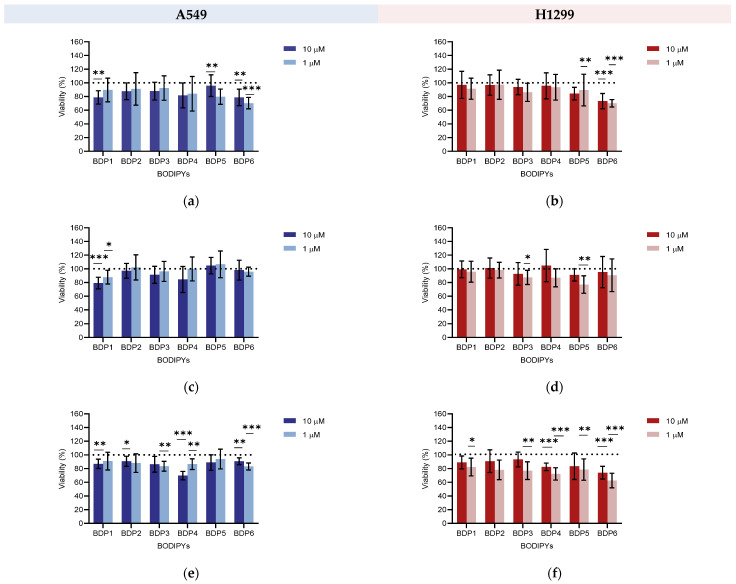
SRB assay results for all BODIPYs (1–6) for cell line A549 in (**a**) 24 h assay, (**c**) 48 h assay, and (**e**) 72 h assay and for cell line H1299 in (**b**) 24 h assay, (**d**) 48 h assay, and (**f**) 72 h assay. The results are presented as the mean and CI of a minimum of three independent assays. Statistically significant differences from the control or between conditions are represented by *, where * means *p* < 0.05, ** means *p* < 0.01, and *** means *p* < 0.001.

The SRB data presented in Figure 9 demonstrate that BODIPYs 5 and 6 reduced the cell density in both cell lines during the 24 and 72 h assays. In contrast, BODIPY 3 emerged as one of the most biocompatible, with only a minor impact in the longer incubation periods. Although it had minimal effects on the metabolic activity of both cell lines at 1 and 10 µM, BODIPY 6 did impact the cellular protein content reduction during the 24 and 72 h experiments. While BODIPY 6 may have disrupted the cell cycle, both cell lines appear to have managed to maintain their protein content and healthy metabolism.

Overall, all BODIPYs resulted in changes in cell viability, with the most remarkable effects becoming more evident in the longer trials. A drug can be considered cytotoxic if the viability values drop below 70% [24]. However, considering that the viability changes observed were at most 30.16% for the A549 line and 37.39% for the H1299 line, it is possible to state that none of the BODIPYs seem to have significant cytotoxic effects on cell viability [21,24]. Based on the MTT and SRB results, BODIPYs 3 and 6 had the most biocompatible results, with metabolic activity above 80% and viability above 70% at concentrations of 10 μM and 1 μM. These two BODIPYs were selected to continue the investigations, allowing us to explore at least two compounds with significantly distinct chemical structures.

**Figure 10:**
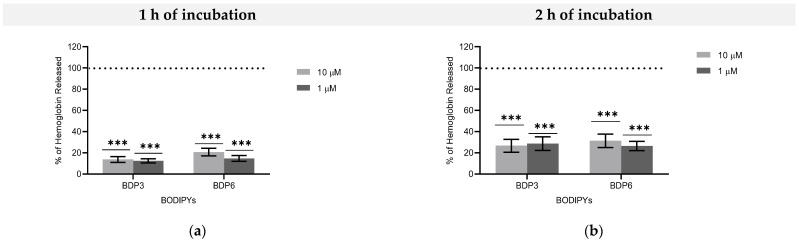
Haemolysis results from (**a**) 1 h of incubation and (**b**) 2 h of incubation of BODIPYs 3 and 6 with red blood cells. The results are presented as the mean and CI of a minimum of three independent assays. Statistically significant differences from the control or between conditions are represented by *** means *p* < 0.001.

Haemolysis is one of the most used methods for evaluating cell toxicity due to the simplicity of isolating erythrocytes, making it a flexible and helpful tool. Figure 10a shows the percentage of haemoglobin released after 1 h of incubation with BODIPYs 3 and 6 at two different concentrations. From the data presented, it is evident that BODIPY 6 had a more considerable detrimental effect on red blood cell membranes. After 2 h of incubation with the two compounds, as shown in Figure 10b, BODIPY 3 triggered a greater release at 10 μM and 1 μM, causing a similar increase in the release when using BODIPY 6 at both concentrations. Both BODIPYs induced greater lysis in the second incubation period.

Comparisons were made considering the maximum release induced by Tryton X-100, the positive control. This surfactant is known to cause damage to the membrane of red cells and can, therefore, be used to assess increased haemoglobin release. Elevated haemoglobin release in the body can have toxic consequences or trigger processes that stress the kidneys or other organs. Therefore, haemolysis is an essential in vitro cytotoxicity assay [25].

The values obtained were encouraging compared to the normalisation value and demonstrated that the compounds appear to have low cytotoxicity. While haemolysis assays are usually conducted with a 1 h incubation period, if a compound tends to cause damage, this should be evident in a shorter period. In this study, we used a 2 h incubation period to try to assess the extent of the effects. Indeed, the values were slightly higher in the second incubation period. According to one article, a formulation is considered haemolytic if it has haemolytic activity above 25%, and the values previously obtained in the first incubation period were below this limit [26]. Even the values obtained in the second incubation period were not considerably above the limit, indicating erythrocyte membrane stability in the presence of BODIPYs [25,26].

**Figure 11:**
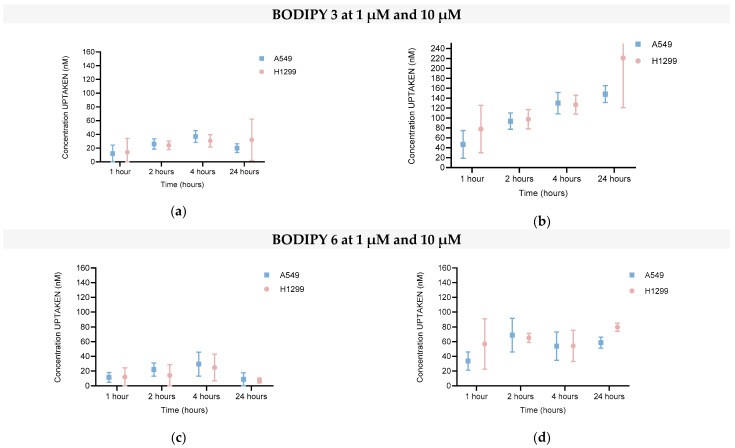
Uptake results (**a**) for 1 μM and (**c**) for 10 μM BODIPY 3 and (**b**) for 1 μM and (**d**) for 10 μM BODIPY 6 for cell lines A549 and H1299, respectively. The results are presented as the mean and CI of a minimum of three independent assays.

The data for BODIPYs 3 and 6 reveal that these compounds are biocompatible with the cell lines and red blood cells. To determine the extent to which BODIPYs 3 and 6 were incorporated by the A549 and H1299 cell lines, a calibration curve was established with a serial dilution of a previously prepared solution of each compound. This procedure is detailed in the Supplementary Information. The cellular uptake assay was conducted over four time periods: 1, 2, 4, and 24 h (Figure 11). These intervals were chosen to assess whether there is a continuous uptake or if an equilibrium is established.

In most of the conditions tested, the uptake values for both compounds in H1299 were lower than those in the A549 cell line, except in the 24 h assay. Since the H1299 cells are derived from a patient who had previously received radiotherapy, it is possible that the cellular mechanisms of compound incorporation have changed or that the BODIPYs face greater difficulty penetrating the cell membranes [27,28]. Nevertheless, the compound incorporation generally increased with longer incubation durations for both cell lines and at all concentrations tested, especially at higher concentrations. It appears that an extended incubation period may be necessary to reach an equilibrium of concentrations inside and outside the cells. This fact may also be related to the drug incorporation mechanism or the compounds’ interactions with the cellular environment. Notably, for the 1 µM concentration, the cells did not exhibit the same trend of increased incorporation with longer assays, suggesting that lower concentrations of the compound might not be incorporated to the same extent in more prolonged assays.

In the literature, an investigation involving a BODIPY for myocardial perfusion imaging evaluated its in vitro uptake after 1.5 h of incubation with 25 µM, yielding incorporation values ranging from 1% to 3% [13]. Despite the modest values achieved in that last investigation, revealing some similarities to the ones that we obtained, an in vivo evaluation of the BODIPY labelled with fluorine-18 indicated its preferential accumulation in the targeted heart tissue [13]. Radiolabelling compounds with fluorine-18 can increase uptake values since fluorine is commonly used to improve the lipophilicity of substances, allowing them to pass more easily through lipid membranes [27,28,29,30]. This aspect should be considered for BODIPYs 3 and 6, as the groups linked to boron could potentially expedite radioactive labelling due to the ring strain on the dialkoxide groups, making them suitable for diagnostic scans.

Given that the BODIPYs under consideration can be radiolabelled and combined with another medicine to achieve a diagnostic and therapeutic ipact, their potential application in the theranostic sector becomes even more intriguing.

## 4. Conclusions

Six different BODIPYs were successfully synthesised and characterised. BODIPYs 1 to 4 showed substantial fluorescence, rendering them promising candidates for dual PET/fluorescence imaging studies. In contrast, BODIPYs 5 and 6 displayed weaker fluorescence, likely attributed to quenching effects triggered by the catechol group covalently linked to the boron atom of BODIPYs.

Incorporating cyclic alkoxides, like catechol derivatives, provides an additional advantageous factor associated with the ring strain on the dialkoxide, which can further accelerate radiolabelling. Based on the structural motif analysis, BODIPYs 5 and 6 have more significant strain regions and greater deviations of boron from the pyrrole plane. Another type of analysis, such as X-ray crystallography or DFT calculations, could be performed to provide us with another perspective on the compounds.

Among the compounds studied, two structures had the most consistent data in the biocompatibility tests. These structures exhibited positive outcomes in both MTT and SRB assays with cancer cells, as well as in subsequent uptake and haemolysis assays. It is worth emphasising that these BODIPYs are characterised by the presence of alkoxyl groups attached to the boron nucleus, contributing to their non-planar structure. This unique structural feature hints at their potential for radiolabelling applications in the future. Following all of the evaluations, BODIPY 3 yielded superior results, while the findings for BODIPY 6 were also noteworthy. An area for improvement to further validate our results could involve increasing the number of samples in the study.

Accurate staging is of paramount importance in predicting prognosis and guiding treatment decisions. Some interesting applications can be mentioned for the compounds studied here. It is worth considering a kinetic assessment of the new alkoxy-BODIPYs compared to their counterparts with ^19^F in the boron centre, as radiolabelling BODIPYs 3 and 6 might enhance their cellular uptake, rendering them suitable for animal studies using EasyPET technology [31]. BODIPY 3 exhibits interesting characteristics for dual-mode imaging, which could open the door for PET/fluorescence-based in vivo investigations. Finally, these compounds might find applications in theranostic approaches that combine PET scans and phototherapy or other therapeutic interventions.

## Figures and Tables

**Table 1:** N-B-N angles obtained from MM2 molecular mechanics calculations.

Structure	N-B-N Angle (°)	Out-of-Plane Deviation (Å)	C-C-O Angle (°)
BODIPY 1	107.27	0.059	-
BODIPY 2	106.77	0.310	-
BODIPY 3	109.50	0.036	121.61
BODIPY 4	108.84	0.063	121.73
BODIPY 5	106.47	0.719	112.37
BODIPY 6	105.15	0.612	112.35

## Data Availability

The data presented in this study are available in the manuscript.

## Supplementary Materials

The following supporting information can be downloaded at https://www.mdpi.com/article/10.3390/ma16227085/s1, Figure S1: ^1^H NMR spectrum of BODIPY 1 (400 MHz, CDCl_3_). Figure S2: ^13^C NMR spectrum of BODIPY 1 (100 MHz, CDCl_3_). Figure S3: ^11^B NMR spectrum of BODIPY 1 (128 MHz, CDCl_3_). Figure S4: ^19^F NMR spectrum of BODIPY 1 (376 MHz, CDCl_3_). Figure S5: Mass spectrum (positive mode) of BODIPY 1. Figure S6: ^1^H NMR spectrum of BODIPY 2 (400 MHz, CDCl_3_). Figure S7: ^13^C NMR spectrum of BODIPY 2 (100 MHz, CDCl_3_). Figure S8: ^11^B NMR spectrum of BODIPY 2 (128 MHz, CDCl_3_). Figure S9: ^19^F NMR spectrum of BODIPY 2 (376 MHz, CDCl_3_). Figure S10: Mass spectrum (positive mode) of BODIPY 2. Figure S11: ^1^H NMR spectrum of BODIPY 3 (400 MHz, CDCl_3_). Figure S12: ^13^C NMR spectrum of BODIPY 3 (100 MHz, CDCl_3_). Figure S13: ^11^B NMR spectrum of BODIPY 3 (128 MHz, CDCl_3_). Figure S14: ^19^F NMR spectrum of BODIPY 3 (376 MHz, CDCl_3_). Figure S15: Mass spectrum (positive mode) of BODIPY 3. Figure S16: ^1^H NMR spectrum of BODIPY 4 (400 MHz, CDCl_3_). Figure S17: ^13^C NMR spectrum of BODIPY 4 (100 MHz, CDCl_3_). Figure S18: ^11^B NMR spectrum of BODIPY 4 (128 MHz, CDCl_3_). Figure S19: Mass spectrum (positive mode) of BODIPY 4. Figure S20: ^1^H NMR spectrum of BODIPY 5 (400 MHz, CDCl_3_). Figure S21: ^13^C NMR spectrum of BODIPY 5 (100 MHz, CDCl_3_). Figure S22: ^11^B NMR spectrum of BODIPY 5 (128 MHz, CDCl_3_). Figure S23: ^19^F NMR spectrum of BODIPY 5 (376, MHz, CDCl_3_). Figure S24: Mass spectrum (positive mode) of BODIPY 5. Figure S25: ^1^H NMR spectrum of BODIPY 6 (400 MHz, CDCl_3_). Figure S26: ^13^C NMR spectrum of BODIPY 6 (100 MHz, CDCl_3_). Figure S27: ^11^B NMR spectrum of BODIPY 6 (128 MHz, CDCl_3_). Figure S28: Mass spectrum (positive mode) of BODIPY 6. Figure S29: Fluorescence intensity spectrum of a dilution series of known concentrations of BODIPY 3 used to obtain the calibration curves on the left side. Moreover, the two calibration curves were obtained with a linear fitting on the right side. The measurement of fluorescence intensity was performed, taking into account the maximum absorption at 545 nm and the fluorescence emission peak at 563 nm. Figure S30: Fluorescence intensity spectrum of a dilution series of known concentrations of BODIPY 6 used to obtain the calibration curves on the left and the two with a linear fitting on the right side. The measurement of fluorescence intensity was performed, taking into account the maximum absorption at 531 nm.
