# Supplementary material for: Synthesis and In Vitro Biocompatibility Studies of Novel Alkoxy 4,4-Difluoro-4-bora-3a,4a-diaza-s-indacenes
Source: Materials (Basel). 2023 Nov 8;16(22):7085. doi: 10.3390/ma16227085 (PMC10672151; doi:10.3390/ma16227085)
Supplement: Supplementary file 1 [file materials-16-07085-s001.zip › materials-2689222-supplementary.pdf]

materials

SUPPLEMENTARY INFORMATION

1) Characterization of BODIPY 1

^{1}H NMR (400 MHz, $CDCl_3$) δ (ppm): 2.54 (s, 6H), 2.33 (q, *J* =7.6 Hz, 4H), 1.51 (s, 6H), 1.02 (t, *J* = 7.6 Hz, 6H).

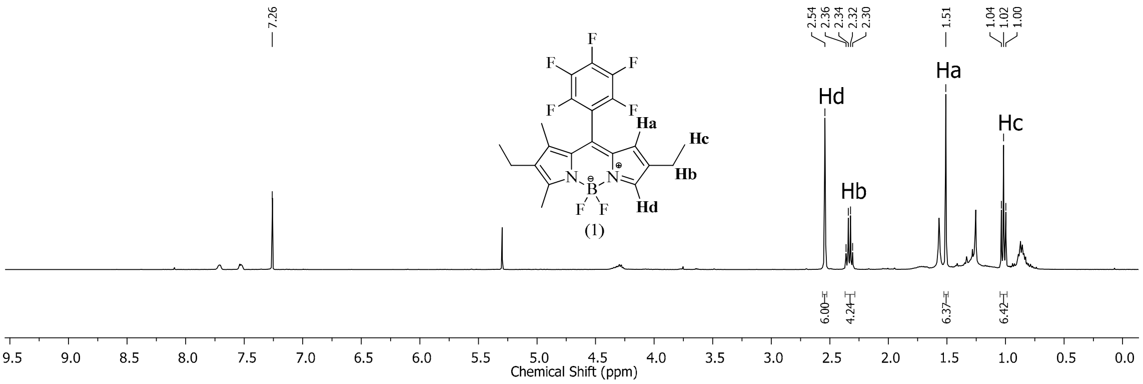

Figure S1 - ^{1}H NMR spectrum of BODIPY 1 (400 MHz, $CDCl_3$).

^{13}C NMR (100 MHz, CDCl3) δ (ppm): 156.14; 136.57; 133.93; 130.91; 130.35; 128.83; 121.16; 110.19; 17.08; 14.53; 12.73; 10.84.

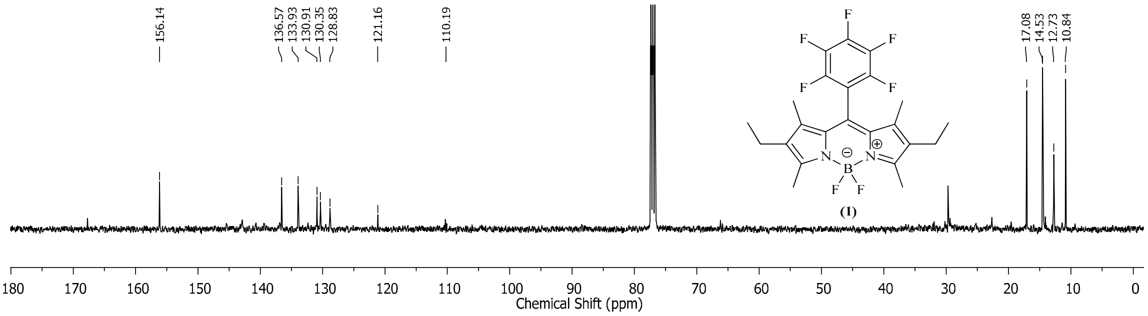

Figure S2 - ^{13}C NMR spectrum of BODIPY 1 (100 MHz, $CDCl_3$).

^{11}B NMR (128 MHz, $CDCl_3$) δ (ppm): 0.70(t, *J* =32.64 Hz).

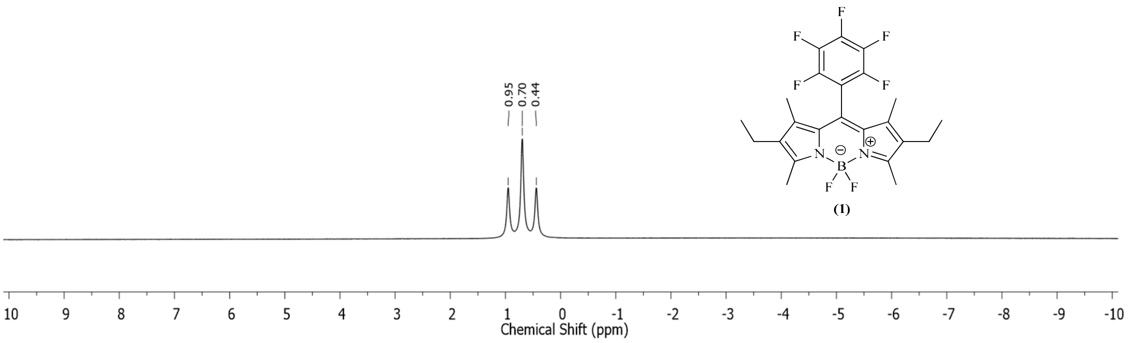

Figure S3 - ^{11}B NMR spectrum of BODIPY 1 (128 MHz, $CDCl_3$).

^{19}F NMR (376 MHz, $CDCl_3$) δ (ppm): -139.25(dd, J = 15.04 Hz, 7.14 Hz, 2F), -145.61 (dd, J = 32.71 Hz, 2F), -151.06 (t, J = 20.68 Hz, F), -159.82 (dt, J = 21.81, 6.40 Hz, 2F).

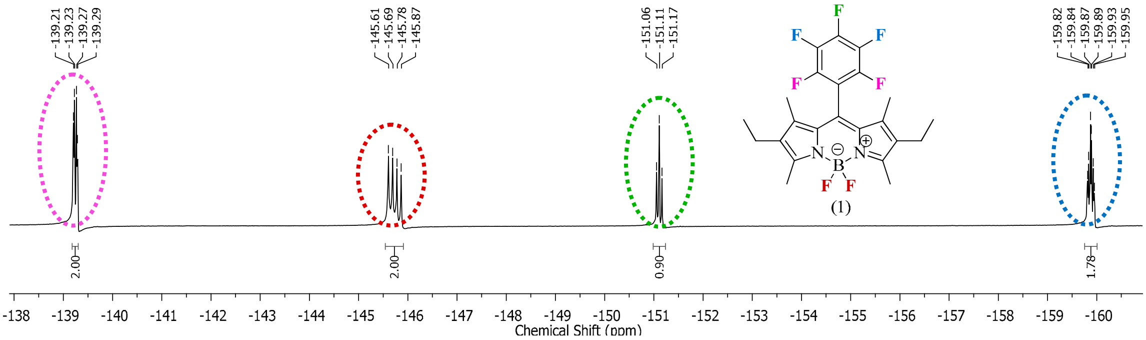

Figure S4 - ^{19}F NMR spectrum of BODIPY 1 (376 MHz, $CDCl_3$).

MS m/z [M+H]⁺ calculated for $C_{23}H_{22}BF_7N_2^+$: 471.23; **Found:** 471.30.

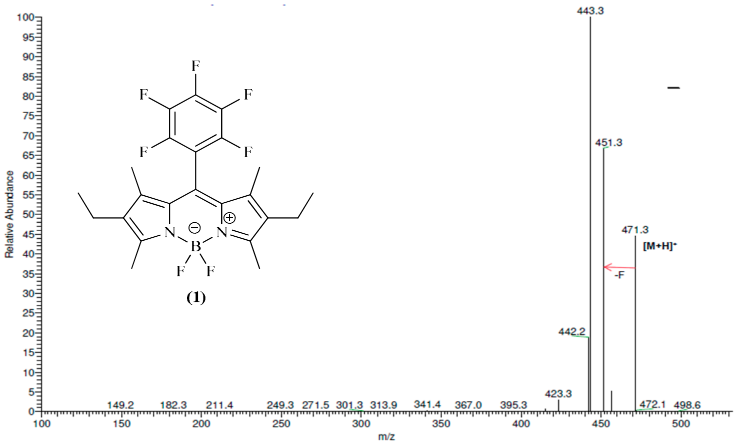

Figure S5 - Mass spectrum (positive mode) of BODIPY 1.

2) Characterization of BODIPY 2

^{1}H NMR (400 MHz, $CDCl_3$) δ (ppm): 7.71-7.70 (m, 2H), 7.54 -7.52 (m, 2H), 2.54 (s, 6H), 2.33 (q, J = 7.6 Hz, 4H), 1.51 (s, 6H), 1.02 (t, J = 7.6 Hz, 6H);

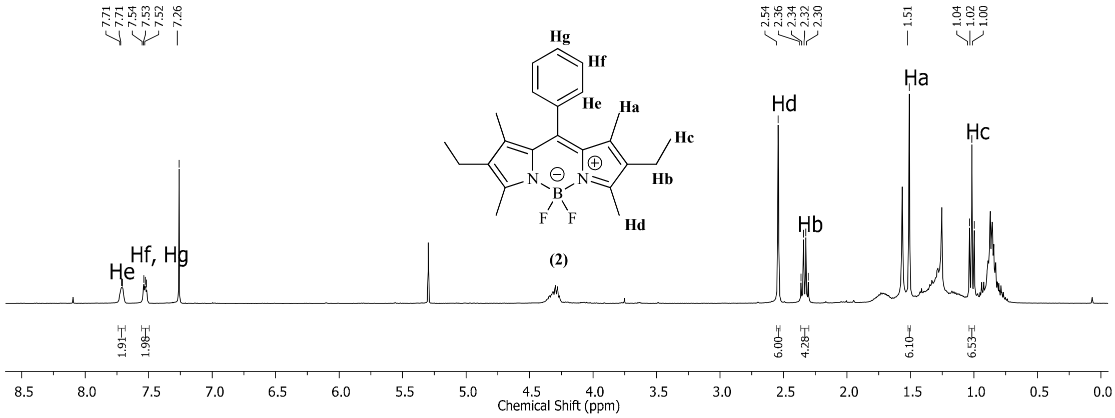

Figure S6 - ^{1}H NMR spectrum of BODIPY 2 (400 MHz, $CDCl_3$).

^{13}**C NMR (100 MHz, $CDCl_3$) δ (ppm)**: 156.14, 136.57, 133.92, 130.91, 128.83, 26.61, 17.09, 14.54, 12.74, 10.85.

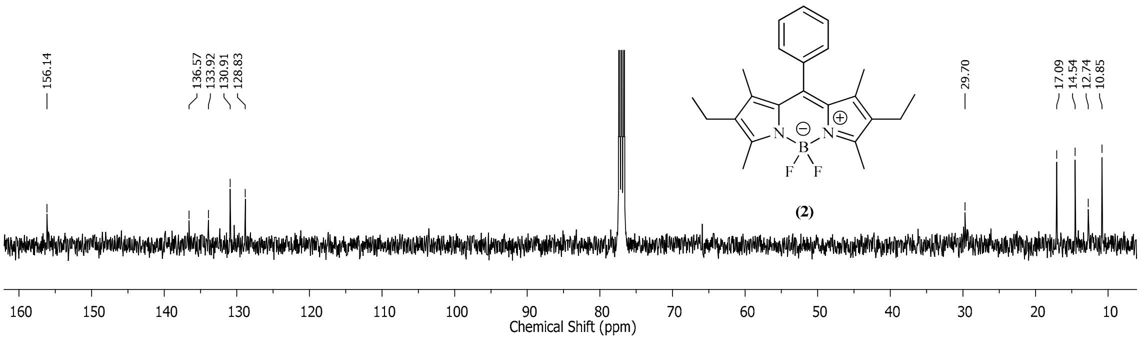

Figure S7 - ^{13}C NMR spectrum of BODIPY 2 (100 MHz, $CDCl_3$).

^{11}**B NMR (128 MHz, CDCl3) δ (ppm):**0.70 (t, *J* =32.64 Hz).

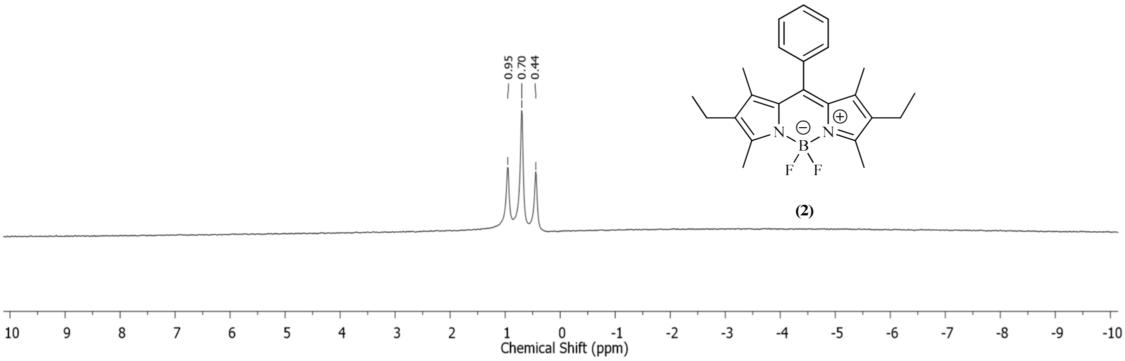

Figure S8 - ^{11}B NMR spectrum of BODIPY 2 (128 MHz, $CDCl_3$).

^{19}**F NMR (376 MHz, $CDCl_3$) δ (ppm):** -145.79 (dd, *J* =33.84 Hz, 2F).

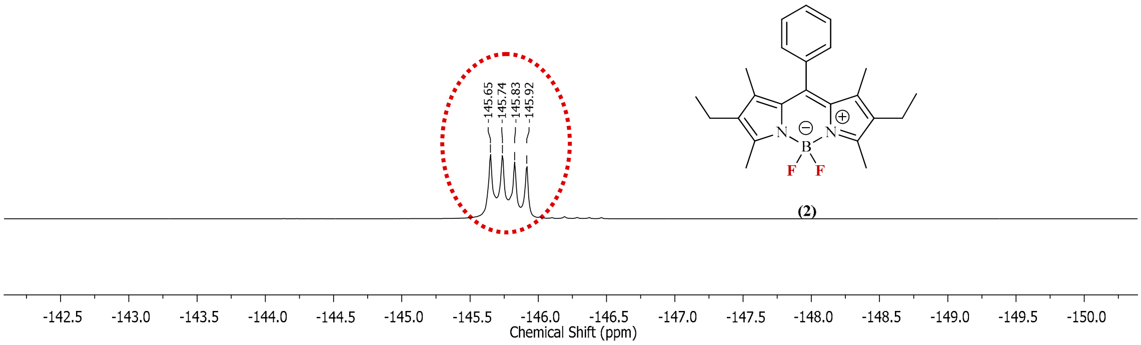

Figure S9 - ^{19}F NMR spectrum of BODIPY 2 (376 MHz, $CDCl_3$).

MS m/z [M+H]$^+$ calculated for $C_{23}H_{28}BF_2N_2^+$: 381.28; **Found:** 381.30.

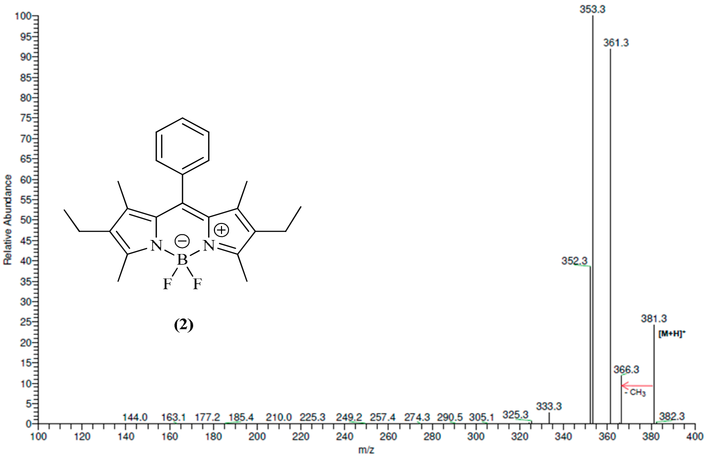

Figure S10 - Mass spectrum (positive mode) of BODIPY 2.

3) Characterization of BODIPY 3

^{1}H NMR (400 MHz, CDCl$_3$) δ (ppm): 7.06 (t, *J* = 7.2 Hz, 4H), 6.76 (t, *J* =7.2 Hz, 2H), 6.65 (d, *J* = 7.6 Hz, 4H), 2.51 (s, 6H), 2.20 (q, *J* = 7.6 Hz, 4H), 1.52 (s, 6H), 0.86 (t, *J* = 7.6 Hz, 6H).

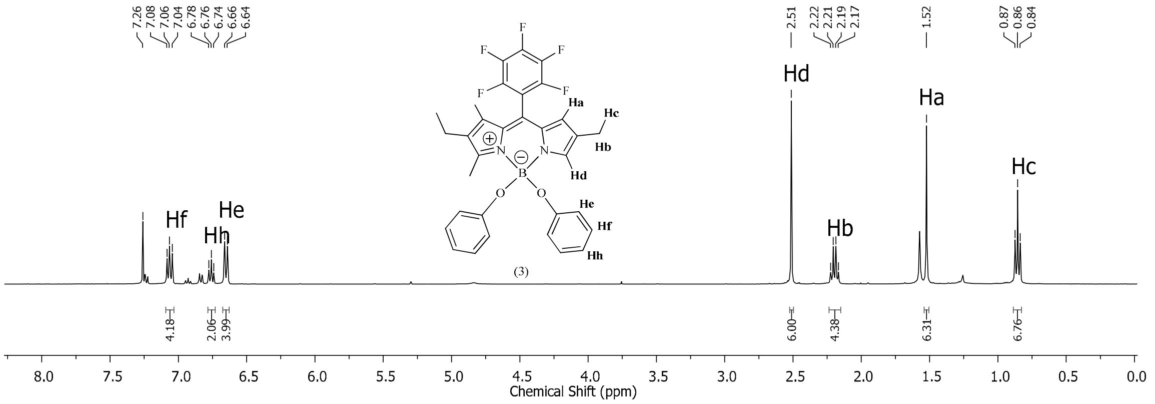

Figure S11 - ^{1}H NMR spectrum of BODIPY 3 (400 MHz, CDCl$_3$).

^{13}C NMR (100 MHz, CDCl$_3$) δ (ppm): 157.01, 156.48, 136.34, 134.50, 130.97, 129.10, 119.50, 118.40, 115.22, 17.08, 14.47, 13.05, 11.02.

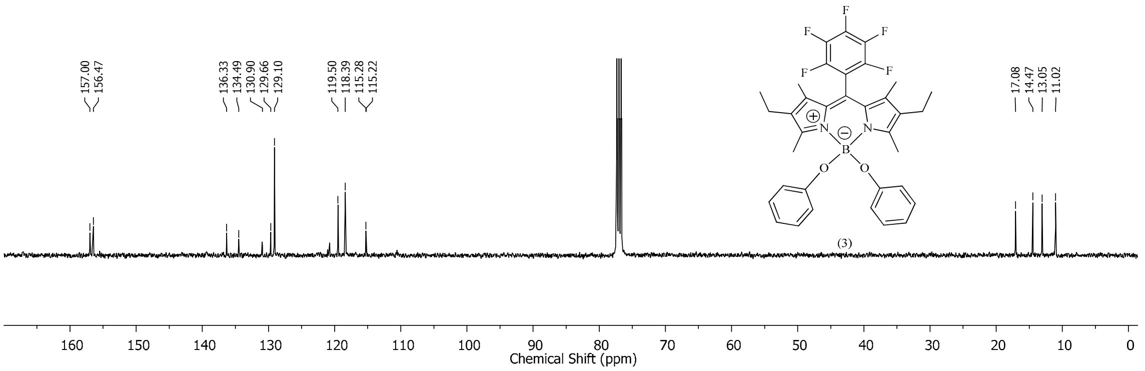

Figure S12 - ^{13}C NMR spectrum of BODIPY 3 (100 MHz, $CDCl_3$).

^{11}B NMR (128 MHz, CDCl3) δ (ppm): 0.83 (s).

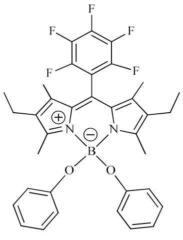

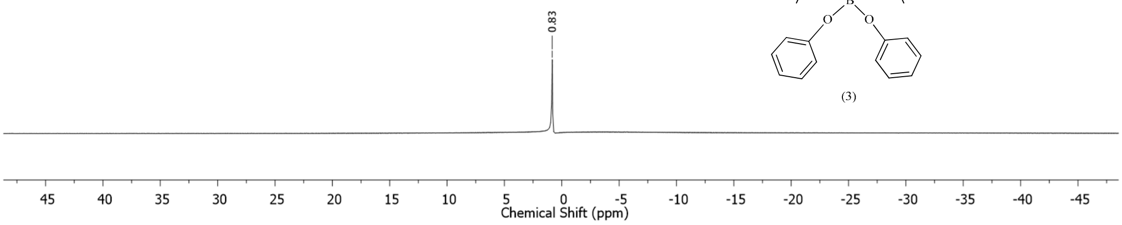

Figure S13 - ^{11}B NMR spectrum of BODIPY 3 (128 MHz, $CDCl_3$).

^{19}F NMR (376 MHz, $CDCl_3$) δ (ppm): -140.08 (dd, *J*= 22.56Hz,7.52Hz, 2F), -151.02 (t, *J*= 20.68Hz, 1F), -159.79 (dt, *J*= 21. 06Hz, 7.52Hz, 2F).

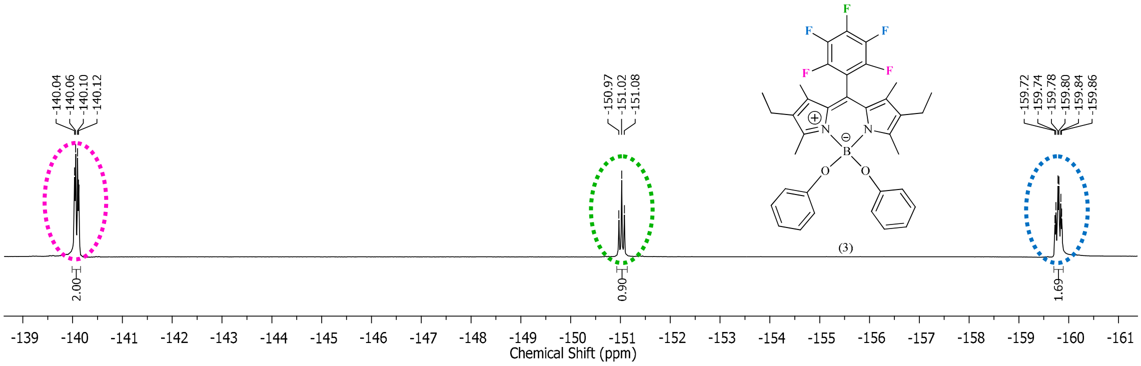

Figure S14 - ^{19}F NMR spectrum of BODIPY 3 (376 MHz, $CDCl_3$).

MS m/z $[M+H]^+$ calculated for $C_{35}H_{33}BF_5N_2O_2^+$: 619.44; **Found:** 641.20.

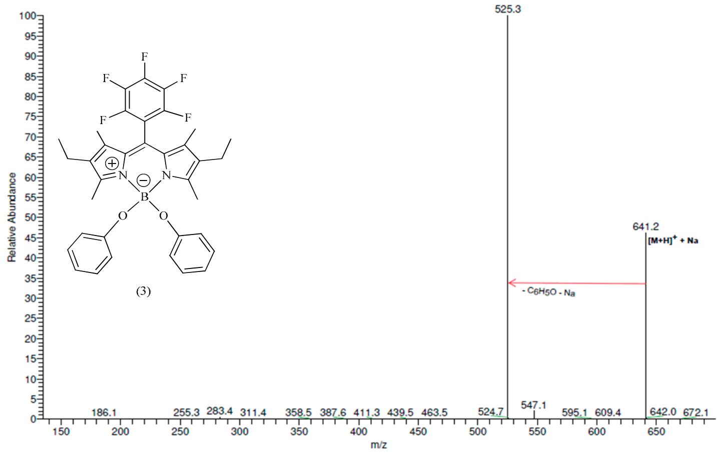

Figure S15 - Mass spectrum (positive mode) of BODIPY 3.

4) Characterization of BODIPY 4

^{1}H NMR (400 MHz, CDCl$_3$) δ (ppm): 7.50 – 7.48 (m, 3H), 7.24 - 7.22 (m, 2H), 7.08 (t, *J* = 7.6 Hz, 4H), 6.77 (t, *J* = 7.2 Hz, 2H), 6.63 (d, *J* = 7.6 Hz, 4H), 2.52 (s, 6H), 2.19 (q, *J* = 7.6 Hz, 4H), 1.26 (s, 6H), 0.85 (t, *J* = 7.6 Hz, 6H).

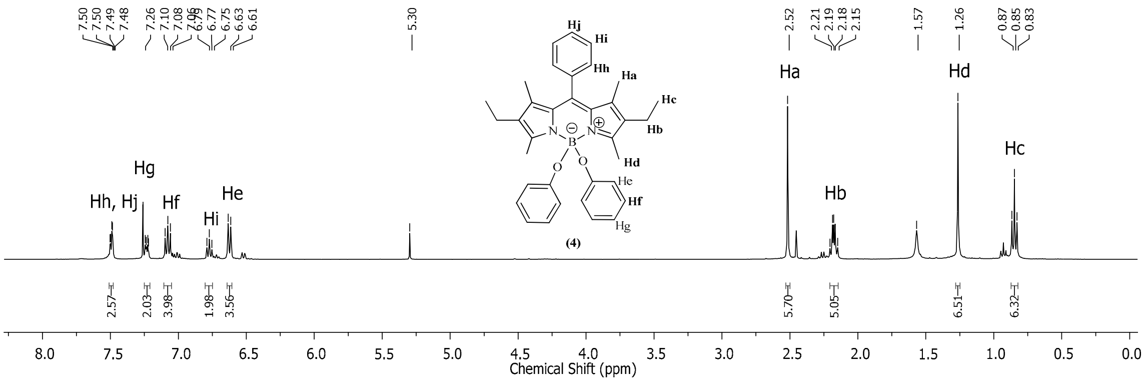

Figure S16 - ^{1}H NMR spectrum of BODIPY 4 (400 MHz, CDCl$_3$).

^{13}C NMR (100 MHz, CDCl3) δ (ppm):156.90, 154.48, 138.10, 136.00, 133.29, 131.38, 128.92, 128.66, 128.39, 119.34, 118.80, 17.05, 14.52, 12.84, 11.73.

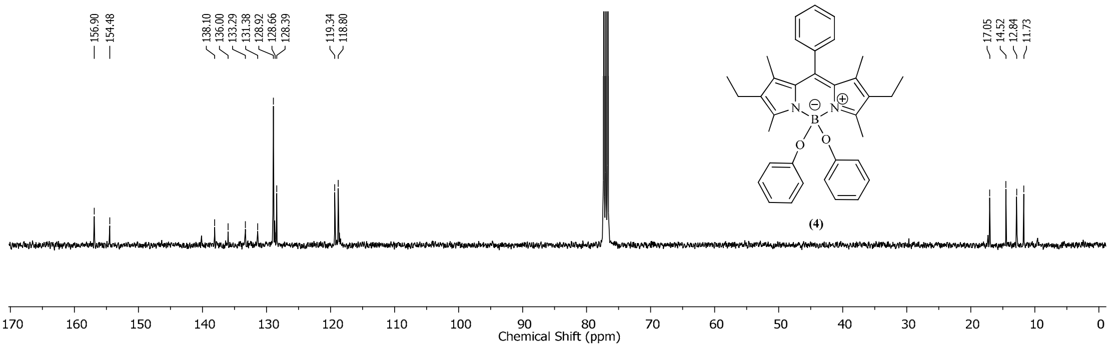

Figure S17 - ^{13}C NMR spectrum of BODIPY 4 (100 MHz, CDCl$_3$).

^{11}B NMR (128 MHz, CDCl3) δ (ppm): 0.91 (s).

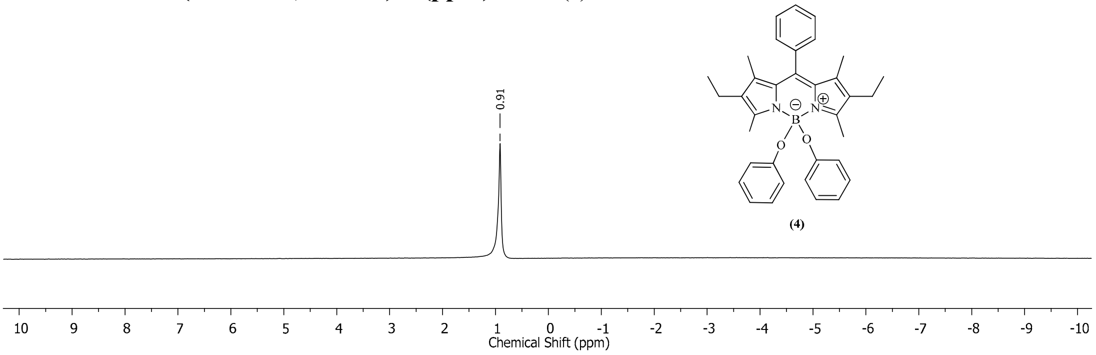

Figure S18 - ^{11}B NMR spectrum of BODIPY 4 (128 MHz, $CDCl_3$).

MS m/z [M+H]$^+$ calculated for $C_{35}H_{38}BN_2O_2^+$: 529.49; **Found**: 551.30.

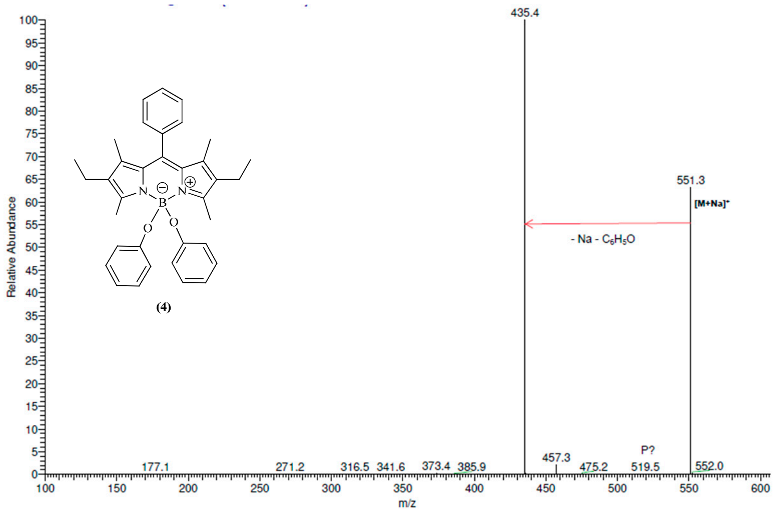

Figure S19 - Mass spectrum (positive mode) of BODIPY 4.

5) Characterization of BODIPY 5

^{1}H NMR (400 MHz, $CDCl_3$) δ (ppm): 6.76 (s, 4H), 2.27 (q, *J* = 7.6 Hz, 4H), 2.05 (s, 6H), 1.51 (s, 6H), 0.96 (t, *J* = 7.6 Hz, 6H).

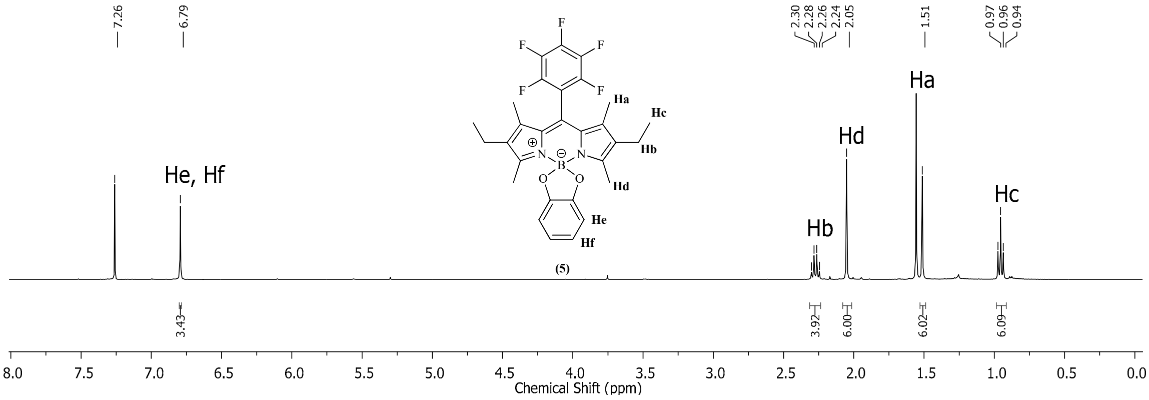

Figure S20 - ^{1}H NMR spectrum of BODIPY 5 (400 MHz, $CDCl_3$).

^{13}C NMR (100 MHz, $CDCl_3$) δ (ppm): 157.99, 151.96, 137.30, 134.57, 131.05, 119.71, 108.84, 17.09, 14.58, 12.92, 11.03.

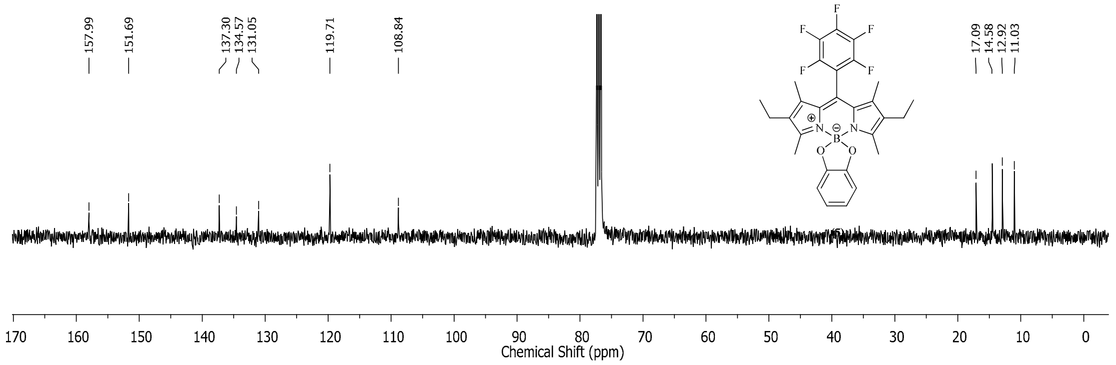

Figure S21 - ^{13}C NMR spectrum of BODIPY 5 (100 MHz, $CDCl_3$).

^{11}B NMR (128 MHz, CDCl3) δ (ppm): 7.15 (s).

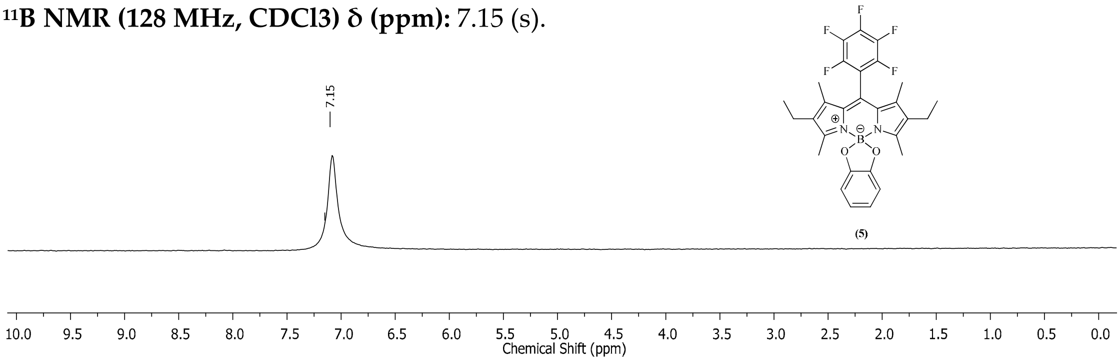

Figure S22 - ^{11}B NMR spectrum of BODIPY 5 (128 MHz, $CDCl_3$).

^{19}F NMR (376 MHz, $CDCl_3$) δ (ppm): -139.16 (dd, *J* = 22.56 Hz, 7.52 Hz, 2F), -151.09 (t, *J* =22.56 Hz, 1F), -159.83 (dt, *J* = 22.56 Hz, 7.52Hz, 2F).

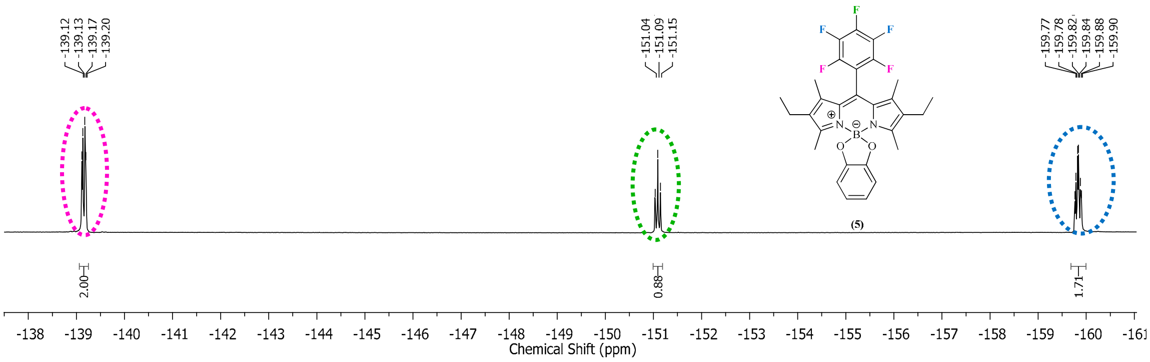

Figure S23 - ^{19}F NMR spectrum of BODIPY 5 (376, MHz, $CDCl_3$)

MS m/z $[M+H]^+$ calculated for $C_{29}H_{27}BF_5N_2O_2^+$: 541.33; **Found:** 541.30.

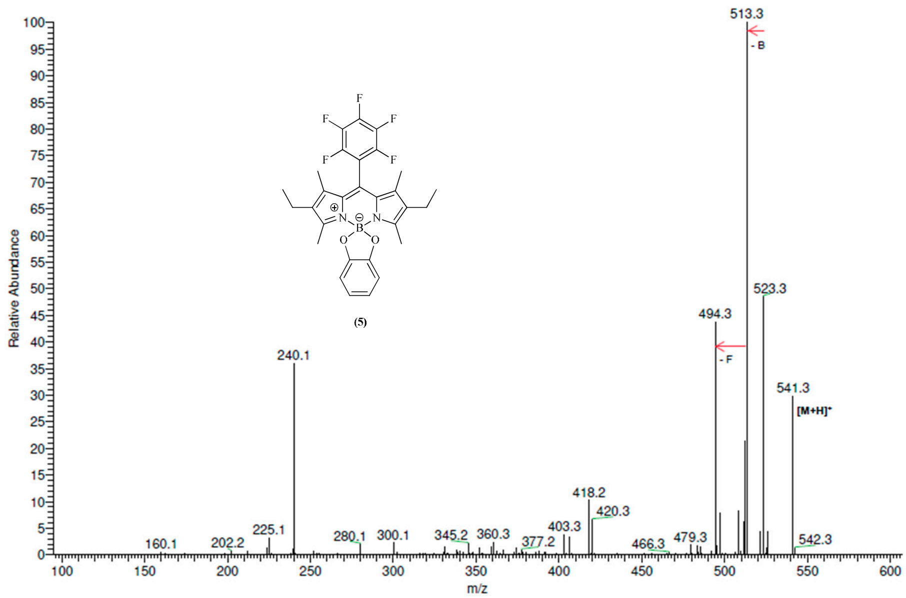

Figure S24 - Mass spectrum (positive mode) of BODIPY 5.

6) Characterization of BODIPY 6

^{1}H NMR (400 MHz, $CDCl_3$) δ (ppm): 7.49-7.47 (m, 3H), 7.31- 7.29 (m, 2H), 6.78 (s, 4H), 2.23 (q, *J* = 7.6 Hz, 4H), 2.24 (s, 6H), 1.27 (s, 6H), 0.91 (t, *J* = 7.6 Hz, 6H).

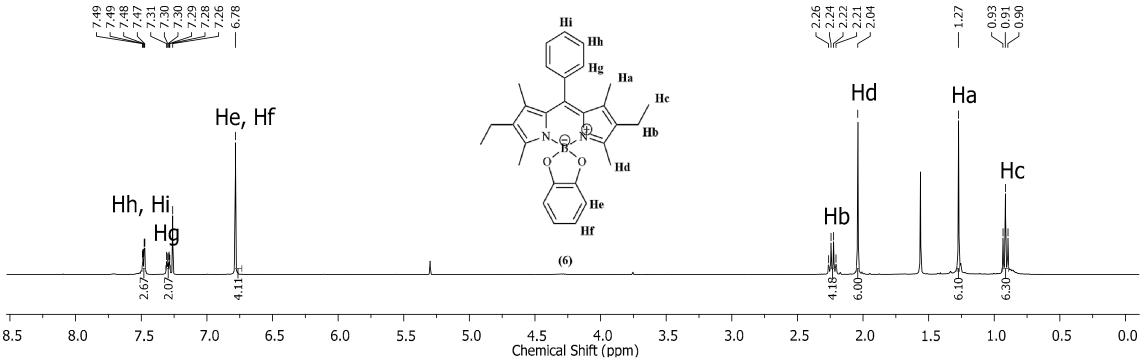

Figure S25 - ^{1}H NMR spectrum of BODIPY 6 (400 MHz, $CDCl_3$).

^{13}C NMR (100 MHz, $CDCl_3$) δ (ppm): 155.60, 155.41, 151.99, 140.04, 139.18, 136.03, 133.40, 131.49, 129.06, 128.73, 128.33, 128.26, 119.39, 108.74, 17.07, 14.60, 12.69, 11.81.

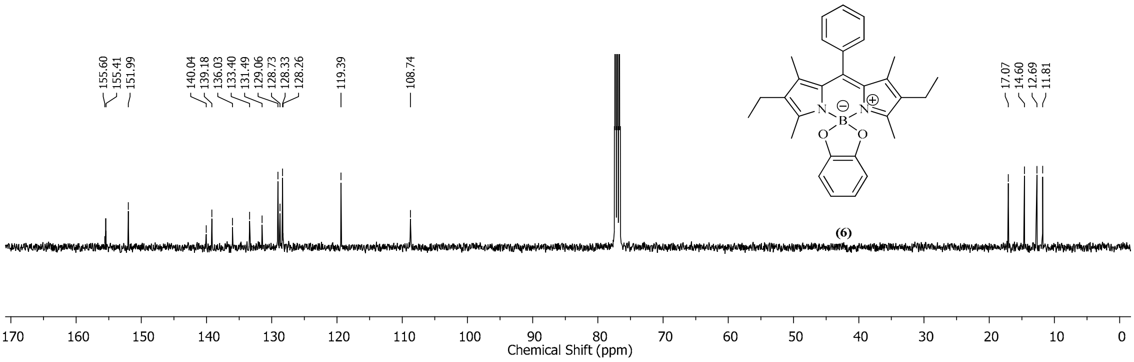

Figure S26 - ^{13}C NMR spectrum of BODIPY 6 (100 MHz, $CDCl_3$).

^{11}B NMR (128 MHz, CDCl3) δ (ppm): 7.14 (s).

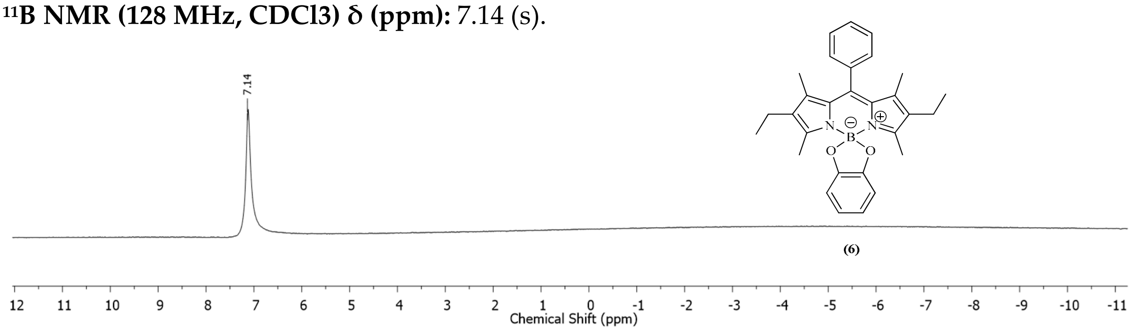

Figure S27 - ^{11}B NMR spectrum of BODIPY 6 (128 MHz, $CDCl_3$).

MS m/z $[M+H]^+$ calculated for $C_{29}H_{32}BN_2O_2^+$: 451.38; **Found:**451.40.

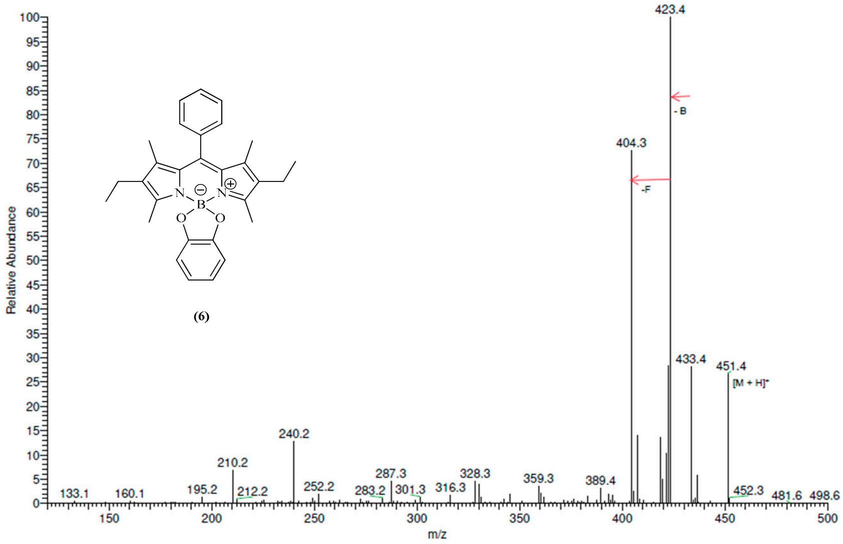

Figure S28 - Mass spectrum (positive mode) of BODIPY 6.

7) UPTAKE Calibration Curves

To obtain the uptake values, it was necessary to determine the calibration curves of BDIPYS 3 and 6. Figures 29 and 30 have associated a graph with data obtained from the measured intensities for each of the different concentrations of the compounds as a function of the range of wavelengths read, and also, the calibration curves obtained by linear fitting the maximum fluorescence as a function of each concentration value. Each calibration curve is associated with the equation from the fitting and the corresponding adjusted R-squared.

As the linear fit model was unsuitable for the entire data set, the data were divided into two groups, and the linear fit was performed for both sets, obtaining two R squares better than the R square obtained for the whole dataset.

The following linear fit equations were used to calculate the concentrations of BODIPY 3 internalized by the cells, represented in the A and B graphs of Figure 14. In the following equations, F represents the fluorescence intensity, and C represents the concentration in nanomolar.

$$F = 962.47C + 4949.78 \text{ for F between } [0, 160\,000] \qquad \text{(equation 4)}$$

$$F = 196.75C + 150305 \text{ for F between }]160\,000, 360\,000] \qquad \text{(equation 5)}$$

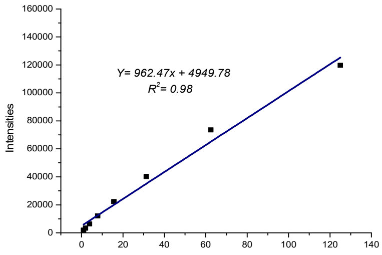

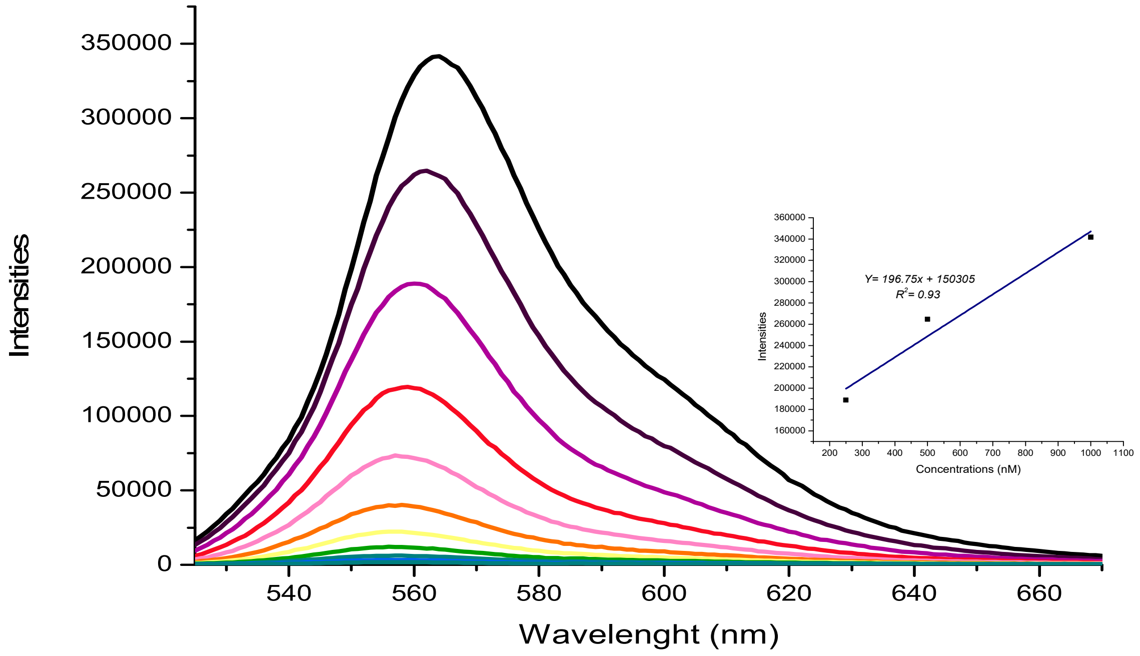

Figure S**29** - Fluorescence intensity spectrum of a dilution series of known concentrations of BODIPY 3 used to obtain the calibration curves on the left side. Moreover, the two calibration curves were obtained with a linear fitting on the right side. The measurement of fluorescence intensity was performed, taking into account the maximum absorption at 545 nm and the fluorescence emission peak at 563 nm.

The plot of intensities as a function of wavelength for BODIPY 6 depicted in Figure 30 suggests that this compound has a very weak fluorescence compared to the graph in Figure 29. This feature had already been mentioned previously by the characterization data, more specifically by the analysis performed on the data in graph F of Figure 1. Even so, it was possible to determine the amounts of BODIPY 6 incorporated by the cells using equations 5 and 6. However, only concentrations with fluorescence more significant than 500 were considered since the linear fitting could only be made for higher fluorescence values

In the following equations, F represents the fluorescence intensity, and C represents the concentration in nanomolar.

$$F = 24.74C + 356.57 \text{ for F between } [500, 3000] \qquad \text{(equation 6)}$$

$$F = 8.27C + 3481.48 \text{ for F between }]3000, 12000] \qquad \text{(equation 7)}$$

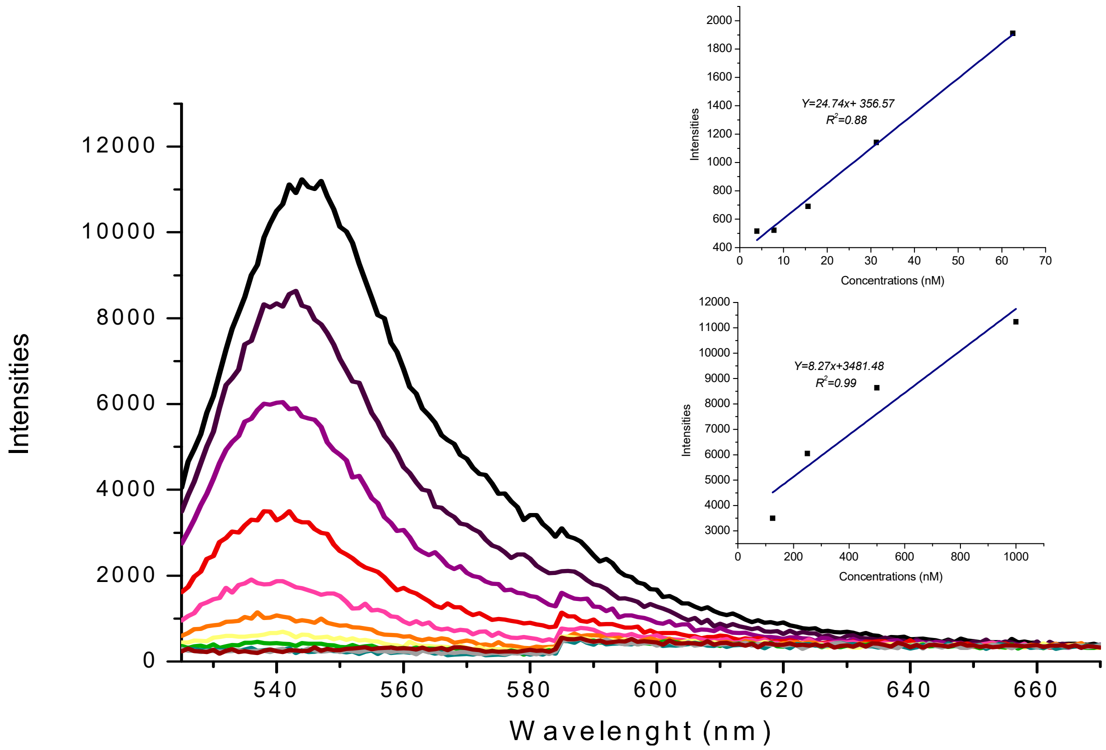

Figure S30 - Fluorescence intensity spectrum of a dilution series of known concentrations of BODIPY 6 used to obtain the calibration curves on the left and the two with a linear fitting on the right side. The measurement of fluorescence intensity was performed, taking into account the maximum absorption at 531 nm.
